# Development of Novel Immobilized Copper–Ligand Complex for Click Chemistry of Biomolecules

**DOI:** 10.3390/molecules29092148

**Published:** 2024-05-05

**Authors:** Rene Kandler, Yomal Benaragama, Manoranjan Bera, Caroline Wang, Rasheda Aktar Samiha, W. M. C. Sameera, Samir Das, Arundhati Nag

**Affiliations:** 1 Carlson School of Chemistry and Biochemistry, Clark University, Worcester, MA 01610, USA; rkandler@clarku.edu (R.K.); mbera@clarku.edu (M.B.); cawang@clarku.edu (C.W.); rsamiha@clarku.edu (R.A.S.); 2 Department of Chemistry, University of Colombo, Colombo 00300, Sri Lanka; yb.benaragama@gmail.com (Y.B.); wmcsameera@lowtem.hokudai.ac.jp (W.M.C.S.)

**Keywords:** PNN ligand, Cu(I) complex, “click”, heterogeneous catalysis, deuterated solvent effect

## Abstract

Copper-catalyzed azide–alkyne cycloaddition click (CuAAC) reaction is widely used to synthesize drug candidates and other biomolecule classes. Homogeneous catalysts, which consist of copper coordinated to a ligand framework, have been optimized for high yield and specificity of the CuAAC reaction, but CuAAC reaction with these catalysts requires the addition of a reducing agent and basic conditions, which can complicate some of the desired syntheses. Additionally, removing copper from the synthesized CuAAC-containing biomolecule is necessary for biological applications but inconvenient and requires additional purification steps. We describe here the design and synthesis of a PNN-type pincer ligand complex with copper (I) that stabilizes the copper (I) and, therefore, can act as a CuAAC catalyst without a reducing agent and base under physiologically relevant conditions. This complex was immobilized on two types of resin, and one of the immobilized catalyst forms worked well under aqueous physiological conditions. Minimal copper leaching was observed from the immobilized catalyst, which allowed its use in multiple reaction cycles without the addition of any reducing agent or base and without recharging with copper ion. The mechanism of the catalytic cycle was rationalized by density functional theory (DFT). This catalyst’s utility was demonstrated by synthesizing coumarin derivatives of small molecules such as ferrocene and sugar.

## 1. Introduction

The “click” reaction has revolutionized the fields of chemistry and chemical biology, and in recognition of that, the 2022 Nobel Prize in Chemistry was given to the pioneers of click chemistry, Professor Carolyn R. Bertozzi, Professor Morten Meldal and Professor K. Barry Sharpless. The Bio-orthogonal “click” reaction has been widely used to probe into cell functions and the discovery of biomolecules, while copper-catalyzed “click” reaction or Cu(I)-catalyzed azide–alkyne cycloaddition (CuAAC) has been used for making molecules of increasing size and complexity, such as pharmaceuticals, biomolecules, and polymers [1]. Monomeric and dimeric copper (I) complexes catalyze the “click” reaction or 1,3-dipolar cycloaddition reaction [2]. Copper can lead to protein denaturation [3] or be associated with biomolecules like proteins and interfere with cellular processes if released from the associated protein [4,5]. The CuAAC reaction is generally carried out under basic conditions since the basic conditions [2] facilitate the removal of the acetylide proton and help generate the intermediate copper acetylide. The base added for CuAAC can compete as a copper ligand and require further purification of the obtained triazoles [6]. Therefore, click reactions of biomolecules typically use strain-promoted azide–alkyne click (SPAAC), and the large, hydrophobic nature of the cyclooctyne group in SPAAC requires the addition of solubilizing moieties, leading to high reagent cost [7].

**Scheme 1:**
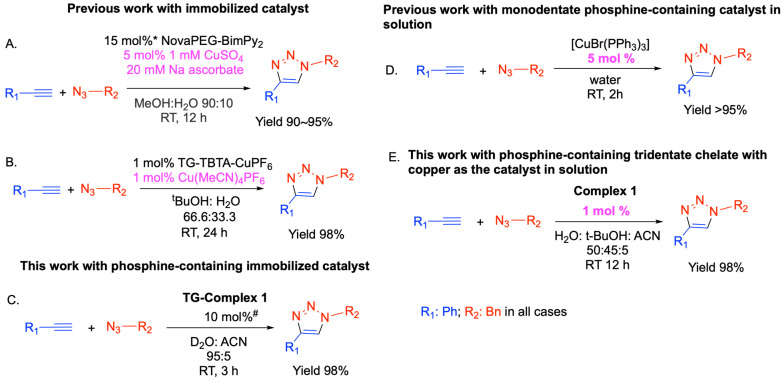
Comparison of this work with previously reported catalysts for CuAAC reaction. (**A**) Fokin et al. [17] utilized additional copper (I) complex in the catalysis reaction. (**B**) Finn et al. [15] used free copper (II) and the reducing agent sodium ascorbate in the catalysis reaction. (**C**) The immobilized complex described in this article catalyzes without free copper and reducing agents. (**D**) Diez-Gonzales et al. [6] used the Cu(I) complex with a monodentate phosphine-containing ligand as a catalyst in solution. (**E**) The developed complex described in this article can work in solution as a CuAAC catalyst at a lower mol%. See Supplementary Materials for the mol percentage calculations of immobilized catalyst (*, #).

An alternative route would be to use the copper catalyst as a heterogeneous catalyst. Copper complexes can function as catalysts in both homogeneous and heterogeneous reactions [8,9]. One of the significant advantages of using heterogeneous catalysts over homogeneous catalysts is the ease of removing copper from the product. Extensive research has been conducted on using tridentate [9] and tetradentate [10] complexes of copper(I) as catalysts for click cycloaddition reactions. However, using these catalysts requires the addition of ascorbic acid as a reducing agent. This is because most tridentate [11] and tetradentate nitrogen donor complexes, such as NNN-type ligands or N4-type ligands [10], by themselves, cannot stabilize the lower Cu(I) oxidation state required for the CuAAC reaction. Adding ascorbic acid can cause side reactions between dehydroascorbate and arginine side chains [12], and the reduction of Cu(II) to Cu(I) by ascorbic acid results in the generation of reactive oxygen species (ROS) that are damaging to biomolecules [13,14]. An ideal heterogenous CuAAC catalyst should be stable and highly efficient and not require the addition of reducing agents like ascorbate or basic conditions to yield the desired products. Finn et al. attempted to develop an efficient heterogeneous CuAAC catalyst by immobilizing tris(benzyltriazolylmethyl)amine (TBTA) framework, an NNN-type ligand, on solid-state resin beads and coordinating copper (Scheme 1A). The resulting complex in the presence of sodium ascorbate as a reducing agent, could be used for multiple catalyst cycles [15]. Other research groups have used a similar approach, immobilized copper complexes with TBTA-like ligands (NNN-type ligand), and used the immobilized catalyst for heterogeneous CuAAC reactions [16,17,18]. However, the reported complexes (Scheme 1B) required the addition of excess copper(I) sources, defeating the intended purpose of using heterogeneous CuAAC catalysts. In this work, our immobilized catalyst (Scheme 1C) did not need an additional Cu(I) source or a reducing agent.

To develop a copper (I) stabilizing ligand framework, we investigated PNN-type complexes, as they can successfully stabilize the Cu(I) oxidation state required for the catalysis, thereby eliminating the need for a reducing agent [19,20]. A subset of PNN-type complexes, copper (I) triphenylphosphine complexes that contain one or two monodentate phosphines and other coligands, have been successfully used as CuAAC catalysts [6,21,22] (Scheme 1D). However, monodentate phosphines are relatively labile and can detach from the complex and react with the azide group to reduce it and form amine [23]. Copper triphenylphosphine compounds with polydentate ligands that stabilize Cu(I) should not experience this issue. We report the development of such a ligand framework with a polydentate phosphine ligand that can stabilize copper (I) without any added base and reducing agent (Scheme 1E). We show that the developed catalyst can be efficiently used for CuAAC reaction with various substrates under mild reaction conditions at room temperature.

## 2. Results and Discussion

### 2.1. Synthesis and Characterization of PNN-Type Complex ***1***

**Figure 1:**
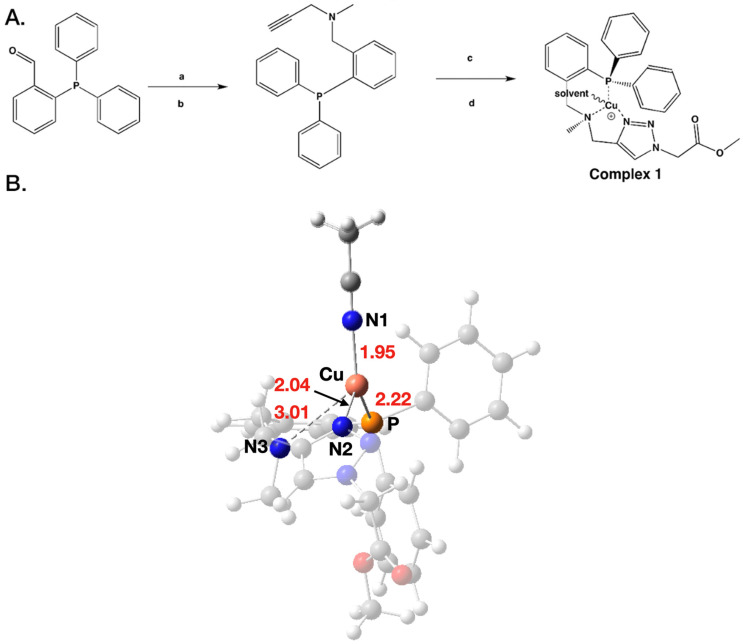
Synthesis and structure of Complex **1**. (**A**) Scheme showing the synthesis of the ligand. (a) Condensation of 2-(diphenylphosphaneyl)benzaldehyde (PBA) to imine with *N*-methyl propargylamine (mPA), MeOH, rt, 52 h, mPA:PBA 5:1. (b) Reduction with NaBH_3_CN (imine:NaBH_3_CN 1:4) pH 5.0–5.5, 3–24 h (c) Complexation of ligand with tetrakis(acetonitrile)copper(I) hexafluorophosphate (1.2:1.0 equivalents) in acetonitrile, 5 h; (d) In-solution click reaction of complexed ligand with methyl 2-azidoacetate, rt, 24 h. (**B**) Optimized structure of Complex **1**, employing DFT. In the optimized structure, Cu (shown in coral) is coordinated with nitrogens N1, N2, N3 (blue), and phosphorus P (orange). Oxygens in the ligand framework are shown in red, while nitrogens are denoted in blue. Cu, N1, N2, and P are in the same plane. Cu-N1 (1.95 Å) and Cu-N1 (2.04 Å) bond distances are relatively shorter compared to Cu-N3 (3.01 Å).

We initially focused on making a PNN-type Schiff’s base complex containing triazole and phosphine, as copper (I) can be stabilized by the imine in Schiff’s base and by the phosphine. However, the complex suffered hydrolysis in the aqueous medium, though it was stable in organic solvents. We reduced the imine to amine to prevent hydrolysis, creating a more flexible sp^3^ nitrogen (Supplementary Materials Figures S-1 and S-2). The resultant PNN-type complex, Complex **1**, was stable in various solvents, including aqueous media. For synthesizing the complex, five equivalents of propargylamine were reacted with one equivalent of 2-(Diphenylphosphanyl)benzaldehyde in methanol for 12 h at room temperature (Figure 1A). The imine reduction was carried out at a low temperature in acidic pH, using four equivalents of sodium cyanoborohydride (NaBH_3_CN) for 12 h and monitored using IR (Supplementary Materials Figure S-3). The reduced ligand was mixed with copper (I) tetrakis (acetonitrile) hexafluorophosphate ([Cu(CH_3_CN)_4_]^+^ PF_6_^−^) at a 1:2.1 ratio in acetonitrile, and the complexation solution after five hours was treated with one-and-half equivalents of methyl-2-azidoacetate, in the presence of four equivalents of *N*,*N*-Diisopropylethylamine (DIEA), to yield Complex **1**. The product was purified by column chromatography with 9:1 hexane:ethyl acetate. NMR and mass spectroscopy were used to characterize the complex in solution (Supplementary Materials Figures S-4 and S-5). Due to the amorphous nature of the catalyst complex, a suitable single crystal for X-ray diffraction studies could not be grown. The energy-minimized structure of the complex, Complex **1**, obtained through density functional theory (DFT) calculations, is shown in Figure 1B (cartesian coordinates listed in Supplementary Materials List S-1). The stability of Complex **1** in acetonitrile was monitored for seven days, and there were no significant changes (Supplementary Materials Figure S-6).

### 2.2. In Solution Catalysis by Complex ***1***

A time course study of triazole by CuAAC reaction catalyzed by Complex **1** was performed using ^1^H NMR spectroscopy. Different solvent compositions were explored for the CuAAC reaction using small molecule substrates, benzyl azide, and phenylacetylene. It was found that the rate and percentage of triazole (Triazole 1) formation (Supplementary Materials Figure S-7) was highly dependent on the solvent compositions. The most effective solvents for the reaction were those that could occupy the fourth coordination position of copper catalyst. Different percentages of water, acetonitrile (ACN), and tertiary butanol (^t^BuOH) were tested as solvents (Table 1), keeping the total volume at 600 μL. For reactions in Table 1, typically 150 mmoles of limiting reagent alkyne and azide were used, except for reaction condition 1G, where lower amounts of reactants were used (50 mmoles) because of their poor water solubilities. The appearance of the triazole adjacent CH_2_ peak at 5.5 ppm was used to quantify the triazole formation (Supplementary Materials Method S-1 or Method S-2). While a long CuAAC reaction in acetonitrile gave a 71.2% yield (condition 1A), the yield was increased slightly to 83.8%, when a mixture of 80:20 ACN:^t^BuOH (condition 1B) was used. Increasing the percentage of tertiary butanol to 50:50 ACN:^t^BuOH increased the conversion rate to 91.5% (condition 1C). The reaction was significantly faster when tertiary butanol was used as the main solvent (95:5 ACN:^t^BuOH) and yielded 95.8% triazole in 48 h (condition 1D). When 20% water (condition E) or 50% water was used (condition 1F), higher conversions were observed, and the reactions were roughly four times faster compared to 5:95 ACN:*^t^*BuOH (condition 1D). The conversion in 90% water was 90.9% after 6 h (condition 1G). Therefore, the reaction rate increased rapidly with a higher percentage of water, which was promising. Percentages of water higher than 90% could not be used for this set of reactants because of their low solubility in water. A control experiment was performed where the solubility of the reactants was high, similar to condition 1F, but utilizing Copper (I) tetrakis (acetonitrile) hexafluorophosphate [Cu(CH_3_CN)_4_]^+^ PF_6_^−^], the precursor copper salt to Complex **1** as the catalyst. The control reaction (condition 1H), when run for twice as long (24 h vs. 12 h), yielded 31.8% product as opposed to 98% yield (condition 1F). This demonstrated that the catalyst efficiency of Complex **1** was responsible for the high yield observed in the CuAAC reaction.

Two other CuAAC reactions were performed to see how the catalyst performed when the reactants were varied, choosing a reaction condition in which the substrates were soluble. Table 2 shows that when the solvent H_2_O/^t^BuOH/ACN was used at a ratio of 45:50:5 for a 12 h reaction of propargyl benzyl ether with benzyl azide to synthesize 1-benzyl-4-(phenoxy methyl)-1*H*-1,2,3-triazole (Triazole 2) (Supplementary Materials Figures S-8 and S-9), the yield of the CuAAC reaction remained high (73.5%) (condition 2A). For another reaction, Complex **1** was used to catalyze the reaction between propargyl-substituted coumarin and benzyl azide to synthesize the therapeutic 4-((1-benzyl-1*H*-1,2,3-triazol-4-yl)methoxy)-2*H*-chromen-2-one (Triazole 3) (Supplementary Materials Figures S-10 and S-11). Propargyl-substituted coumarin had a low solubility in the 50:45:5 mixture of H_2_O/^t^BuOH/ACN; therefore, the solvent mixture ratio was changed to 25:25:50, lowering the percentage of water. This reaction was slower and less efficient, and in 30 h, a yield of 61% was obtained (condition 2B). 

### 2.3. Effect of Catalyst Concentration on Reaction Yield

Next, we studied the effect of varying the amount of catalyst on the yield of the CuAAC reaction under conditions with high percentages of water for the CuAAC reaction yielding Triazole 1 (Table 3). Our goal was to find the catalyst percentage and conditions that are most efficient at high percentages of water and, therefore, more biocompatible. The reactions were completed at a total volume of 600 μL and 50 mmoles of alkyne and azide were used. We found that the reaction yield was highly dependent on the catalyst amount. Thus, using a 2 mol% catalyst led to a 91% yield (condition 3A), while a much lower yield (43%) was observed when using a 1 mol% catalyst (condition 3B). On using a 0.5 mol% catalyst, the yield dropped to 14% (condition 3C).

### 2.4. Effect of Deuterated Water on Reaction Yield

Surprisingly, using deuterated water instead of regular water had a highly positive effect on the reaction yield. Even when using a 1 mol% catalyst, a low-yielding (43%) condition, the use of deuterated water (D_2_O) instead of water (H_2_O) (condition 3D) led to a high yield of 89.2%, compared to a 43% yield (condition 3B). The use of D_2_O even alleviated the effect of using a low mol% of 0.5%, with a high yield of 87.1% observed for the reaction in D_2_O/^t^BuOH/ACN (condition 3E). A deuterated solvent typically shows a slower reaction rate than water (solvent kinetic effect), and this difference is utilized to analyze the kinetics of reactions. However, an inverse solvent kinetic isotope effect (IKIE), in which deuterated solvent accelerates the reaction rate, was observed by Semenov and coworkers. During the monitoring of the autocatalytic CuAAC reaction between tripropargylamine and 2-azidoethanol in the presence of CuSO_4_, it was observed that the generation of the catalyst species took 25 min using a deuterated solvent, D_2_O:MeOD (9:4, *v*:*v*), while the same step using H_2_O:MeOH (9:4, *v*:*v*) under otherwise identical conditions took 114 min. Thus, the deuterated solvent accelerated the generation of the autocatalyst species by 4.56 times. The authors first hypothesized that the IKIE observed was due to the exchange of the alkyne protons of tripropargylamine with deuterium from D_2_O and/or deuterated methanol (MeOD). However, further experiments did not support this hypothesis. The authors speculated that deuterated solvents influenced the rate of reduction of Cu(II) to Cu(I) through an isotope-dependent solvation effect that reduces the activation free energy of electron transfer [24]. To check if additional factors were at play, we checked the pH of a D_2_O-containing reaction and an analogous H_2_O-containing reaction. We found the pH of the former was 6.9, while the pH of the latter was 6.2. Since the copper-catalyzed cycloaddition reaction should work more efficiently at higher pH, the higher pH of a D_2_O-containing reaction could cause a higher yield than the analogous H_2_O-containing reaction. 

### 2.5. Immobilizing Complex ***1*** on Solid Support

**Figure 2:**
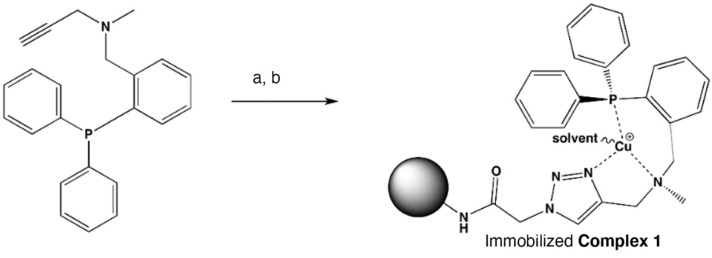
Immobilization of ligand on solid support to yield immobilized Complex **1**. a. Ligand: [Cu(CH_3_CN)_4_]^+^ PF_6_^−^ (1.2:1.0 equivalents) in acetonitrile (ACN), 5 h; b. 1 equivalent ligand and 4 equivalents DIEA, added to 1 equivalent resin-bound azide, rt, 72 h, followed by ACN washes.

Following the characterization and reaction conditions for using Complex **1** as a catalyst, we immobilized it on supports like TentaGel-S-NH_2_, a polyethylene glycol and polystyrene-based resin beads (diameter and capacity 0.31 mmol/g), and on silica-based resin 3-aminopropyl silica (Figure 2). A 25 mmoles amount of benzyl azide and phenylacetylene were used for the reaction. A 10 mg amount (6–13 μM) of silica immobilized Complex **1** and 21.0 mg of Complex **1** immobilized on Tentagel-S-NH_2_ resin were compared. The different amounts of resin were taken to maintain equal equivalents of the catalyst (catalyst concentration around 6 μM) for the reaction, based on the different loading capacity (moles/g) of the two resins.

The reactions were performed in water, tertiary butanol, and acetonitrile at a ratio of 50:45:5 at room temperature (Table 4). The reactions were monitored by ^1^H-NMR up to 120 h to ensure that the reaction was completed. We found a yield of 62.0% for the catalyst on silica and 94.7% for the catalyst on Tentagel resin, respectively. If we compare the yields to the analogous in-solution condition (condition 1F), we see that obtaining the yield comparable to the in-solution condition took much longer for the Tentagel resin. For the silica resin, the yield was much lower even after 120 h. Though silica resin has a higher loading factor of 0.6–1.3 mmol/g, it does not swell in aqueous mixtures, while Tentagel resin swells because of polyethylene glycol. Therefore, the Tentagel resin is soaked in the solvent, and the immobilized catalyst can participate in the reaction. In contrast, the catalyst immobilized on silica resin is not exposed well to the reaction medium. Since Tentagel resin yielded more promising results, further experiments were conducted with Complex **1** immobilized on Tentagel resin.

### 2.6. Effect of Deuterated Solvent and pH Variation on CuAAC Reaction Yield for Solid Immobilized Catalyst

Due to the promising results obtained with deuterated water for the in-solution reactions with Complex **1**, we explored using deuterated water in the reaction with Complex 1 immobilized on solid phase (Tentagel-S-NH_2_ resin) and did reactions for shorter times (Table 5). All reactions were performed with 17.8 mg of resin. For the reaction, 50 mmoles of each alkyne and azide were used and the total volume was maintained at 600 μL. When distilled water with a small percent of acetonitrile (95:5) was used for the reaction solvent mixture, a moderate yield of approximately 50–60 percent was obtained after 12 h (condition 5A). On reducing the reaction time to 3 h, as expected, the yield went down (21%) (condition 5B). When using deuterated water in the solvent mixture (95:5) for the same reaction, a yield of 98% was obtained in 12 h, showing a significantly higher yield and a 10× faster reaction rate (condition 5C). Even more impressive was that when the reaction was performed for 3 h under the same conditions, a high yield of 98% was observed, indicating a 40× faster reaction rate (condition 5D). To determine if the difference in pH was responsible for this significant reaction rate acceleration, a third set of experiments was performed where the pH of the distilled water was adjusted to 7.0. This increased the 12 h reaction yield to around 90–98% (condition 5E), compared to 50–60% observed previously with non-deuterated water (condition 4A). At the higher pH of 7, the yield for a 3 h reaction also improved significantly, to 54–64% (condition 5F) from 21% (condition 5B), though it was not as efficient as the reaction with the deuterated solvent (condition 5D). Therefore, while the pH of the reaction played a significant role in its reaction rate and yield, the pH was not the only factor. The accelerated reaction rate observed in the deuterated solvent was partly due to the change in pH and partly due to the isotope-dependent solvation effect [24].

### 2.7. Efficiency of CuAAC Reactions with Complex ***1*** Catalyst Immobilized on the Solid Support

**Figure 3:**
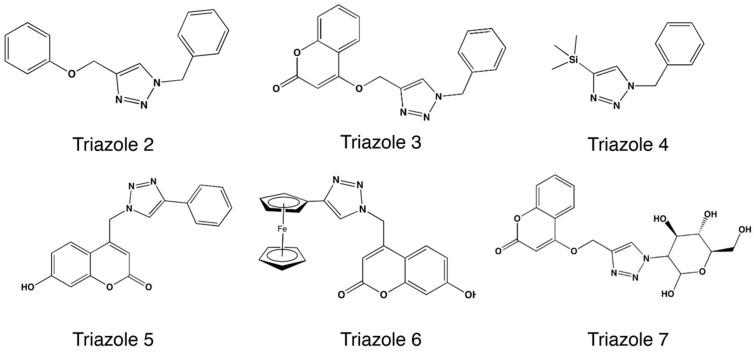
Various triazoles synthesized using immobilized Complex **1**, in addition to Triazole 1.

To determine the efficiency of immobilized Complex **1** to act as a catalyst for CuAAC reaction using different substrates, a series of CuAAC reactions were performed, yielding the triazoles shown in Figure 3. For each reaction, 50 mmoles of alkyne and azide were used, and the total volume was kept constant at 600 μL. 17.8 mg amount of Tentagel-S-NH_2_ resin with immobilized Complex **1** was used. A mixture of deuterated water with ^t^BuOH and ACN was used to study the CuAAC reactions with different reactants (Table 6). Using a solvent mixture of D_2_O:t-BuOH:ACN at a ratio of 50:45:5 gave high yields for Triazole 2 and Triazole 3 (98.1% and 90%, respectively) (conditions 6A and 6C). The yield of Triazole 1 using 95:5 deuterated water with acetonitrile also gave a high yield of 98%. (condition 6B). When distilled water was used for the CuAAC reaction to yield Triazole 3, 4-((1-benzyl-1*H*-1,2,3-triazol-4-yl)methoxy)-2*H*-chromen-2-one, the yield was much lower (67.4%) after a 12 h CuAAC reaction in H_2_O:t-BuOH:ACN in the ratio 25:25:50 (condition 6D). Replacing the water with deuterated water increased the reaction rate 4 times, like what was observed for Triazole 1, and gave a yield of 77.5% after a 3 h CuAAC reaction (condition 6E). The yield of Triazole 4 (Supplementary Materials Figures S-12 and S-13) after a 12 h reaction in D_2_O/ACN (95:5) was 54.5%, because of the volatility of the silane starting material (condition 6F). For the synthesis of Triazole 5, 7-hydroxy-4-((4-phenyl-1*H*-1,2,3-triazol-1-yl)methyl)-2*H*-chromen-2-one, (Supplementary Materials Figures S-14 and S-15), we observed a close to complete conversion (98%) after 24 h in ACN:^t^BuOH:D_2_O (33.33:33.33:33.33) (condition 6G) (Supplementary Materials Method S-3, Figure S-16).

We also made coumarin containing triazoles 4-ferrocene-((1*H*-1,2,3-triazol-1-yl)methyl)-7-hydroxy-2*H*-chromen-2-one (Triazole 6) and a mixture of (3*R*,4*S*,5*R*)-3,4,5,6-tetrahydroxy-2-(4-(((2-oxo-2*H*-chromen-4-yl)oxy)methyl)-1*H*-1,2,3-triazol-1-yl)hexanal with 4-((1-((4*R*,5*S*,6*R*)-2,4,5-trihydroxy-6-(hydroxymethyl)tetrahydro-2*H*-pyran-3-yl)-1*H*-1,2,3-triazol-4-yl)methoxy)-2*H*-chromen-2-one (Triazole 7) by CuAAC reaction with Complex **1** immobilized on Tentagel resin as catalyst. New triazole derivatives such as Triazole 6 and Triazole 7 formed by combining coumarin with ferrocene or D-deoxy glucose, show potential for anion labeling [25], may have anticancer [26,27] and antimicrobial [28] properties and can be used for glycolysis inhibition [29] and imaging in cancer cells [30]. The yield of Triazole 6 (Supplementary Materials Figures S-17 and S-18) was 43% after 24 h in a D_2_O/^t^BuOH/ACN (33.33:33.33:33.33) solvent mixture (condition 6H). The synthesis of Triazole 7 at room temperature (condition 6I) had a low yield of 25%. To obtain a reasonable yield of Triazole 7, the temperature was increased to 37 °C, and the reaction was performed for a longer time of 48 h in D_2_O:^t^BuOH:ACN (25:25:50). Triazole 7 was purified and characterized (Supplementary Materials Figures S-20–S-22) when a 79% yield of Triazole 7 was observed (condition 6J) (Supplementary Materials Method S-4, Figure S-22).

### 2.8. Stability of the Immobilized Complex ***1*** Catalyst

Reusing immobilized Complex **1** as a catalyst was studied for the CuAAC reaction yielding Triazole 3. The CuAAC reaction yielding Triazole 3 was chosen for determining the performance of immobilized Complex **1** over multiple cycles, as the Triazole 3 product was more soluble compared to other triazole products under the reaction conditions. Monitoring other CUAAC reactions multiple times while recovering the bead-bound catalyst from the reaction without any loss of beads was rendered difficult by the precipitation of the products and their association with the resin-bound catalyst. 

**Figure 4:**
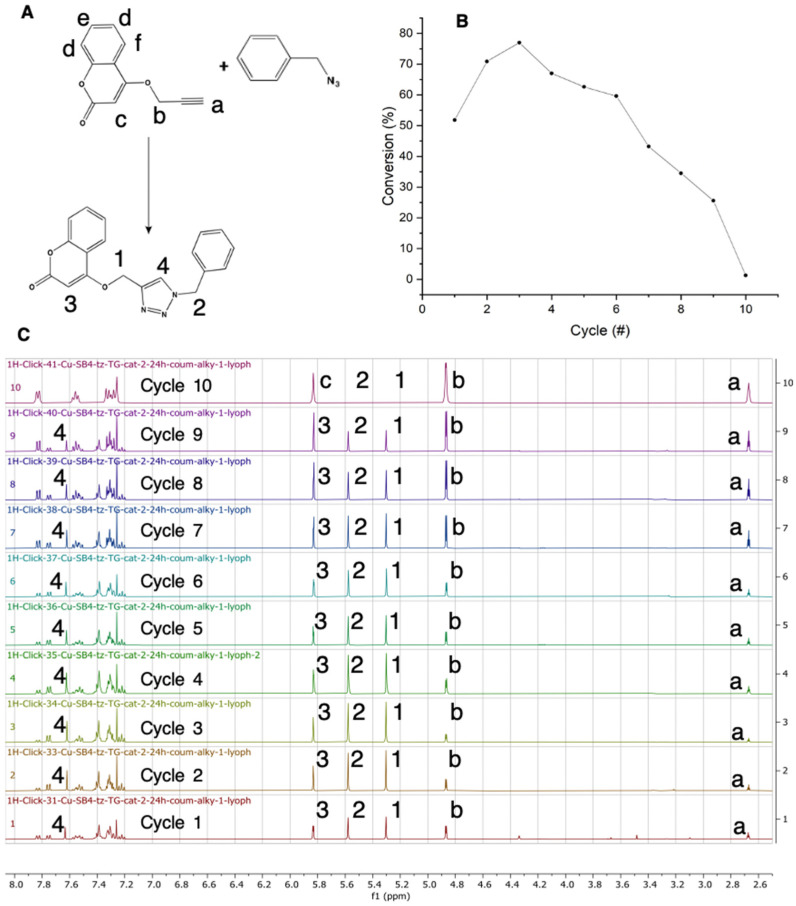
Repeated use of immobilized Complex **1** for the CuAAC reaction yields Triazole 3 without recharging the complex with copper. (**A**) Reaction scheme and labeled protons in alkyne and triazole; a–f refer to protons of the starting material alkyne, and 1–4 refer to the protons of the product triazole. (**B**) Percentage of Triazole 3 formation in cycles 1–10; (**C**) ^1^H-NMR of cycles 1–10.

Unlike previously reported immobilized complexes, the immobilized Complex **1** could be reused multiple times without recharging the catalyst with copper (Figure 4). The first catalysis cycle had a lower conversion to Triazole 3 (52%). Cycles 2–6 showed robust conversions to Triazole 4 (65–77% in 24 h). While theoretically, the CuAAC reaction mechanism could proceed both via mononuclear and binuclear copper complex-mediated catalysis, it has been demonstrated that there are two equivalent copper centers in the intermediate of the reaction, in which the second copper atom acts as a stabilizing donor ligand to a highly energetic and unstable mononuclear metallacycle intermediate [31]. We hypothesized that during the first cycle, the copper complexes on the beads were required to orient with respect to each other to facilitate the formation of the binuclear intermediate. This orientation was already present in the second and third cycles, so the yield was higher than in the first cycle. The conversions in additional cycles decreased gradually. After ten cycles, there was no significant conversion to triazole product (1%). We monitored copper leaching using a standard colorimetric test [32]. A basic solution of sodium diethyldithiocarbamate, which has a darkening yellow color in the presence of copper, was used to wash the immobilized complex, and the washes were analyzed by monitoring the absorbance at 437 nm. We generated a standard calibration curve (Supplementary Materials Figure S-23) for the copper diethyldithiocarbamate solutions. Using this calibration curve, we could quantify the copper leaching from the resin by quantifying the amount of copper diethyldithiocarbamate complex in the washes. Previous (less efficient) versions of catalysts before Complex **1** (such as the non-reduced imine complex mentioned in Section 2.1) showed a quantifiable amount of copper diethyldithiocarbamate after synthesis (Supplementary Materials Figure S-23). However, for Complex **1**, the leaching was so minimal that we could not quantify any copper diethyldithiocarbamate in the wash solutions after the Complex **1** synthesis using the colorimetric assay, indicating minimal leaching. This was promising, given that another reported immobilized CuAAC catalyst had to have a copper source, such as [Cu(MeCN)_4_]PF_6_ in the reaction, to undergo successive catalysis cycles [17]. While another immobilized CuAAC catalyst worked for seven subsequent cycles without recharging copper and had activity similar to the catalyst reported here, it required the addition of 10 mM Na ascorbate for each CuAAC reaction and was performed in 9:1 MeOH:H_2_O [15] and so occurred in a much higher percentage of organic solvent than the reported conditions in this article.

### 2.9. Computed Mechanism of the Catalytic Cycle

**Figure 5:**
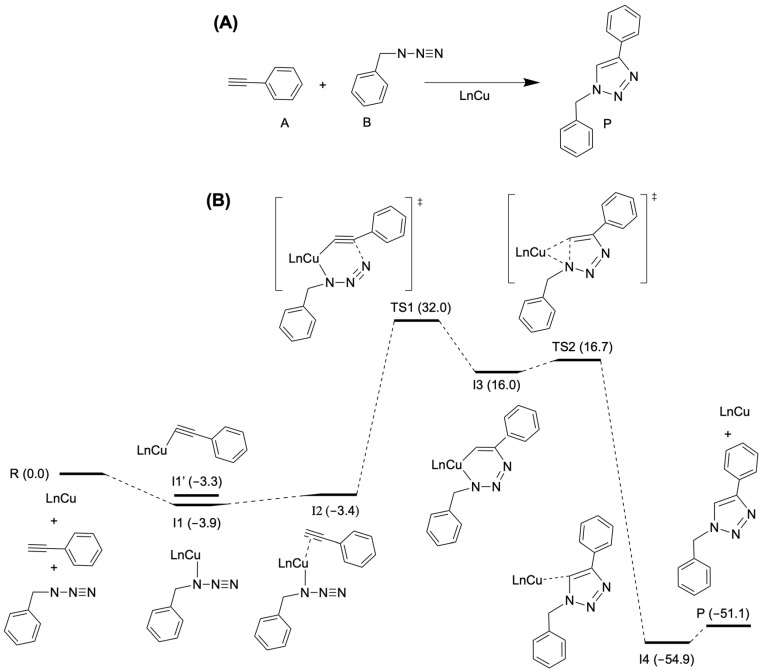
(**A**) Reaction used for the mechanistic study; (**B**) free energy (kcal/mol) profile of the mechanism of the catalytic cycle.

We have used the reaction shown in Figure 5A for the computational mechanistic survey. The computed free energy profile for the mechanism of the catalytic cycle is shown in Figure 5B. The sum of the free energy of isolated A, B, and LnCu was used as the reference energy point. A or B could coordinate to LnCu, and the resulting complexes were 3.3 (I1′) and 3.9 (I1) kcal/mol stable relative to the entry point of the free energy profile. To initiate the cyclization process, both A and B must coordinate to Cu, and the resulting complex, I2 (−3.4 kcal/mol), was energetically similar to I1 and I1′. Thus, I1, I1′, and I2 could be formed in solution. The cyclization was initiated through the N-C bond formation, and this step had a free energy barrier of 35.4 kcal/mol (TS1), giving rise to I3. After that, cyclization was completed through the C-C bond formation, and this step had a free energy barrier of 0.7 kcal/mol (TS2). The product of the reaction P was still at the metal coordination sphere of the subsequent intermediate, I4. Then, the product could be separated from the catalyst, and this step only required 3.8 kcal/mol. The rate-determining of the reaction was the initial N-C bond formation (TS1). (Co-ordinates given in Supplementary Materials List S-2).

## 3. Experimental Section

### 3.1. Synthesis of Phosphine Ligand

To synthesize *N*-(2-diphenylphosphaneyl)benzylidine)prop-2-yn-1–aminium, a round bottom flask was flushed with argon and covered with aluminum foil to protect it from light. About 50 mL of methanol was purged with argon for 5 min. A 5:1 mixture of 5 mmol propargylamine and 1 mmol 2-(Diphenylphosphanyl)benzaldehyde was used for the reaction. The 2-(Diphenylphosphanyl)benzaldehyde was dissolved in 50 mL methanol, and a methanol solution of propargylamine was added. The yellow solution was stirred for 12 h at room temperature until the solution had changed to orange. The reaction was monitored by FT-IR (functional group transformation of carboxyl of aldehyde (1695 cm^−1^) to imine (1640 cm^−1^)) for up to 12 h. 

The dissolved imine from the 1st step was kept on ice for 30 min before the pH adjustment. The pH was measured after adding acetic acid and the reducing agent (4 eq. NaBH_3_CN) for 12 h. After removing the solvent under vacuum, the product was extracted with DCM and filtered. The remaining precipitate (mainly reducing agent) was washed with DCM until the solution remained colorless (color change in DCM solution from orange to yellow to colorless). The orange and yellow solutions were combined. The DCM was removed by vacuum, and the product was purified by silica gel column chromatography (silica gel 0.035–0.070 mm, 60 Å) with 9:1 hexane:ethyl acetate. The collected fractions were monitored by TLC (9:1, hexane:ethyl acetate (R_f_ 0.575). The purified ligand was analyzed by LC-MS, IR, and ^1^H, ^13^C NMR. The overall yield was 11.5% after column chromatography. 

### 3.2. Analysis of Ligand

ESI-MS [M+H]^+^ found *m*/*z* = 328.1 calculated value for [C_22_H_20_NP]^+^ = 328.1.

^1^H NMR (400 MHz, CDCl_3_) δ 9.22 (dt, *J* = 4.9, 2.0 Hz, 1 H), 8.01 (ddd, *J* = 7.8, 3.9, 1.5 Hz, 1 H), 7.42–7.36 (m, 1 H), 7.36–7.26 (m, 12 H), 6.90 (ddd, *J* = 7.7, 4.7, 1.4 Hz, 1 H), 4.37 (t, *J* = 2.2 Hz, 2 H), 2.46–2.33 (m, 1 H).

^13^C NMR (101 MHz, CDCl_3_) δ 161.35, 161.13, 139.06, 138.89, 138.23, 138.04, 136.31, 136.22, 134.14, 134.05, 133.94, 133.85, 133.34, 132.04, 131.95, 130.75, 130.56, 127.53, 127.48, 78.87, 75.43, 47.10.

### 3.3. Synthesis of Complex

For the complexation; a 1.2:1 mixture of the ligand (55.2 mg; 0.193 mmol; 1.2 eq.) and [Cu(CH_3_CN)_4_]^+^ PF_6_^−^ (50 mg; 0.161 mmol; 1 eq.) was prepared by dissolving them individually in acetonitrile (ACN). The orange–red ligand solution and colorless copper (I) solution were transferred to a round-bottom flask. After 5 h; DIEA (4 eq.; 0.643 mmol; 113.3 μL) and methyl-2-azidoacetate (1.5 eq.; 0.241 mmol; 24.7 μL) were added to the solution. The solution was stirred for 24 h. The solution was transferred, and the solvent was removed by vacuum. 

ESI-MS [M]^+^ found *m*/*z* = 521.1 calculated value for [C_26_H_27_CuN_4_O_2_P]^+^ 521.1; [M+(C_3_H_8_O)_2_]^+^ found *m*/*z* = 642.1, calculated value for [C_26_H_27_CuN_4_O_2_P^+^(C_3_H_8_O)_2_]^+^ 642.2.

^1^H NMR (400 MHz, CD_3_CN) δ 7.82 (s, 1 H), 7.54–7.48 (m, 3 H), 7.48–7.40 (m, 13 H), 7.38 (dd, *J* = 7.5, 1.3 Hz, 1 H), 7.36–7.30 (m, 4 H), 6.94 (dd, *J* = 8.7, 7.5, 1.3 Hz, 1 H), 5.26 (s, 2 H), 3.95 (s, 1 H), 3.89 (s, 3 H), 3.76 (s, 1 H), 3.46 (s, 2 H), 2.47 (s, 3 H).

^13^C NMR (101 MHz, CD_3_CN) δ 166.83, 145.14, 139.90, 139.73, 134.04, 133.95, 133.72, 133.56, 133.12, 131.81, 131.53, 130.99, 130.57, 130.48, 129.22, 129.17, 129.13, 129.03, 124.15, 62.84, 62.77, 54.27, 52.67, 52.03, 51.29, 49.76, 44.24.

### 3.4. Complexation with [Cu(CH_3_CN)_4_]^+^ PF_6_^−^ and Immobilization of Ligand to Form Immobilized Complex ***1***

For the complexation, a 1.2:1 equivalent mixture of ligand and [Cu(CH_3_CN)_4_]^+^ PF_6_^−^ were prepared by first dissolving them individually in ACN. The orange–red ligand and colorless copper (I) solution were transferred to a round-bottom flask and stirred for 5 h. 

The loading capacity of a resin depends on its nature and can also vary slightly from batch to batch. We used TentaGel-S-NH_2_ resin (TG) with a loading capacity 0.29 mmol/g and silica resin (Si) with a loading capacity 1.3 mmol/g. The two resins were treated with 4 equivalents of azidoacetic acid, 3.98 equivalents of 2-(1H-7-Azabenzotriazol-1-yl)-1,1,3,3-tetramethyl uronium hexafluorophosphate (HATU) and 12 equivalents of N,N-Diisopropylethylamine (DIEA), to generate the corresponding azide functionalized resins.

The complexation solution and 4 equivalents of DIEA were added to each azide functionalized resin in a 10 mL solid phase extraction (SPE) tube and reacted for 72 h. The immobilized Complex **1** was washed with ACN, methanol, and DCM and dried. The immobilized resin was stored at −20 °C.

### 3.5. Computational Methods

Geometry optimization was performed using density functional theory (DFT), as implemented in Gaussian16 [33] program. The B3LYP [34,35,36] functional, including empirical dispersion corrections [37,38] were used. The SDD [39,40] basis set and associated effective core potential were applied for Cu, and the 6–31 G(d) [41,42,43] basis sets were used for other atoms. The polarizable continuum model (PCM) [44] was used as the implicit solvation model, where acetonitrile was employed as the solvent. The potential energy of the optimized structures was computed as the single-point energy, employing the SDD basis set for Cu and the cc-pVTZ [45] basis set for other atoms.

## 4. Conclusions

We have developed a catalyst, Complex **1**, that can catalyze the CUAAC reaction without any reducing agent and base. We demonstrated that the catalyst could be used for homogeneous and heterogeneous reactions and give high yields for the CuAAC reactions. Both homogeneous and heterogeneous catalysts performed better in deuterated water than in water. The immobilized catalyst was successfully used to make potential therapeutic and labeling agents like 4-((1-benzyl-1*H*-1,2,3-triazol-4-yl)methoxy)-2*H*-chromen-2-one) (77.5% yield) and 7-hydroxy-4-((4-phenyl-1*H*-1,2,3-triazol-1-yl)methyl)-2*H*-chromen-2-one with high conversion (98% yield). While we have not explored the scalability of this method, a similar approach of immobilizing a CuAAC catalyst on resin [15] has been (Scheme 1B) performed on a preparatory scale and then used to make a series of glycomimetic ligands for the human asialoglycoprotein receptor protein [46]. We anticipate we can do a similar scale-up of the immobilized Complex **1** without any issues and use the catalyst to generate a variety of biologically relevant small molecule “click” products. This catalyst performed moderately well for seven cycles without a reducing agent or base and without recharging copper ions. So, Complex **1** can be used in minute quantity during protein screening under physiological conditions, to induce the formation of a 1,4-substituted 1,2,3-triazole between a ligand containing an alkyne and a protein containing an azide [47]. 

## Figures and Tables

**Table 1:** CuAAC reaction catalyzed by Complex **1** using different solvents.

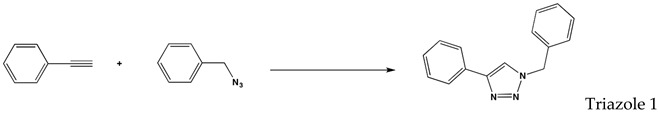				
Entry	Solvent	Composition % (*v*:*v*:*v*)	Time (h)	Conversion (%)
A	ACN	100	114	71.5
B	ACN:H_2_O	80:20	120	83.8
C	ACN:^t^BuOH	50:50	120	91.5
D	ACN:^t^BuOH	5:95	48	95.8
E	H_2_O:^t^BuOH:ACN	20:75:5	12	93.5
F	H_2_O:^t^BuOH:ACN	50:45:5	12	98.1
G	H_2_O:^t^BuOH:ACN	90:5:5	6	90.9
H *	H_2_O:^t^BuOH:ACN	50:45:5	24	31.8

* Control reaction [Cu(CH_3_CN)_4_]^+^ PF_6_^−^], used instead of complex **1** as the catalyst.

**Table 2:** CuAAC reaction with Complex **1** as a catalyst using different substrates.

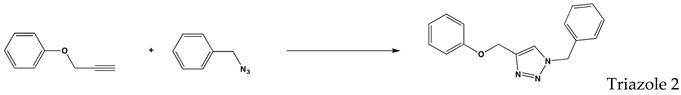				
**Condition**	**Solvent**	**Composition** **% (*v*:*v*:*v*)**	**Time** **(h)**	**Conversion (%)**
A	H_2_O:^t^BuOH:ACN	45:50:5	12	73.5
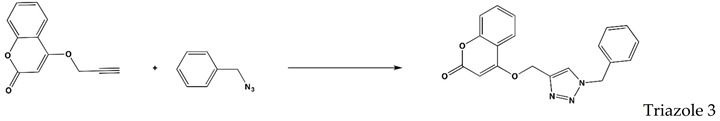				
**Condition**	**Solvent**	**Composition** **% (*v*:*v*:*v*)**	**Time** **(h)**	**Conversion (%)**
B	H_2_O:^t^BuOH:ACN	25:25:50	30	61.0

**Table 3:** Effect of varying amounts of catalyst on yield of CuAAC reaction.

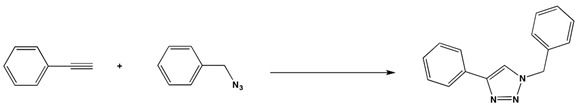				
Condition	Solvent (mol% Catalyst)	Composition % (*v*:*v*:*v*)	Time (h)	Conversion (%)
A	H_2_O:^t^BuOH:ACN (2)	90:5:5	6	90.9
B	H_2_O:^t^BuOH:ACN (1)	90:5:5	6	43.0
C	H_2_O:^t^BuOH:ACN (0.5)	90:5:5	6	13.6
D	D_2_O:^t^BuOH:ACN (1)	90:5:5	6	89.2
E	D_2_O:^t^BuOH:ACN (0.5)	90:5:5	6	87.1

**Table 4:** Performance of immobilized catalyst in solution vs. on solid support.

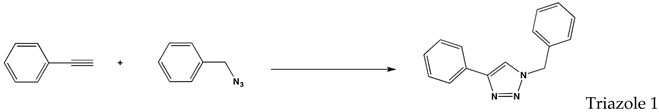				
Condition	Solvent	Composition % (*v*:*v*:*v*)	Time (h)	Conversion (%)
On silica	H_2_O:^t^BuOH:ACN	50:45:5	120	62.0
On Tentagel	H_2_O:^t^BuOH:ACN	50:45:5	120	94.7

**Table 5:** CuAAC reaction with immobilized Complex **1** with water and deuterated water under different pH conditions.

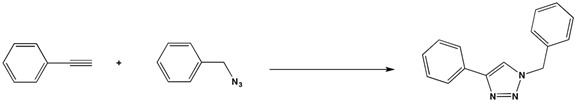					
Condition	Solvent	Composition % (*v*:*v*:*v*)	Time (h)	Conversion (%)	pH
A	H_2_O:^t^BuOH	95:5	12	59.3 ^a^/52.1 ^b^	6.2
B	H_2_O:^t^BuOH	95:5	3	21.0 ^b^	6.2
C	D_2_O:^t^BuOH	95:5	12	98.0	6.9
D	D_2_O:^t^BuOH	95:5	3	98.0 ^a^/98.0 ^b^	6.9
E	H_2_O:^t^BuOH	95:5	12	98.0 ^a^/90.0 ^b^	7.0
F	H_2_O:^t^BuOH	95:5	3	63.6 ^a^/58.7 ^b,c^	7.0

^a^ Percentage yield calculated using Supplementary Materials Method S-1, by comparing triazole protons to the remaining terminal proton of the alkyne. ^b^ Determined by comparison of the aromatic peaks and the average of the normalized triazole integrals at 8.4, 7.75, and 5.5 in the dissolved sample using Supplementary Materials Method S-2. ^c^ Correlation of the three triazole integrals at 8.4 ppm (integral 1.0), 7.75 ppm (2.0), and 5.5 ppm (2.0) was off in this sample. The standard deviation was high, with 6.8% (method a) and 17.9% (method b).

**Table 6:** Optimized conditions for the formation of triazoles by CuAAC using Complex **1** immobilized on Tentagel resin.

Condition	Product	Solvent	Composition % (*v*:*v*:*v*)	Time (h)	Conversion (%)
A	Triazole 1	D_2_O:t-BuOH:ACN	50:45:5	12	98.1
B	Triazole 1	D_2_O:ACN	95:5	3	98.0
C	Triazole 2	D_2_O:^t^BuOH:ACN	50:45:5	12	90.0
D	Triazole 3	H_2_O:^t^BuOH:ACN	25:25:50	24	67.4
E	Triazole 3	D_2_O:^t^BuOH:ACN	25:25:50	3	77.5
F	Triazole 4	D_2_O:ACN	95:5	12	54.5
G ^a^	Triazole 5	D_2_O:^t^BuOH:ACN	33.33:33:33.33	24	98.0
H	Triazole 6	D_2_O:^t^BuOH:ACN	33.33:33:33.33	24	43.0
I ^b,c^	Triazole 7	D_2_O:^t^BuOH:ACN	25:25:50	24	24.5
J ^b,c^	Triazole 7	D_2_O:^t^BuOH:ACN	25:25:50	48 *	78.9

^a^ The percentage yield calculation is described in Supplementary Materials Method S-3. ^b^ The percentage yield calculation is described in Supplementary Materials Method S-4. * Temperature 37 °C; ^c^ mixture of two triazole products, see Supplementary Materials Figure S-19.

## Data Availability

Data are contained within the article and Supplementary Materials.

## Supplementary Materials

The following supporting information can be downloaded at: https://www.mdpi.com/article/10.3390/molecules29092148/s1, Figure S-1, ^1^H NMR of N-(2-diphenylphosphaneyl)benzylidine)prop-2-yn-1-aminium, Figure S-2, MS of reduced and purified ligand, Figure S-3: Monitoring the formation of Complex **1** by FT-IR, Figure S-4, ESI-MS of Complex **1**, Figure S-5, NMR of Complex **1**, List S-1: Cartesian coordinates of the optimized structure of Complex **1**, Figure S-6, Stability of Complex **1** in solution, Figure S-7, LC-MS of Triazole 1, Method S-1, Quantification of CuAAC reaction yield from NMR, Method S-2, Monitoring of Triazole 1 formation in solutions with 50% or more water content, Figure S-8, ^1^H NMR of Triazole 2 in CDCl_3_, Figure S-9, LC-MS characterization of Triazole 2, Figure S-10, ^1^H NMR of Triazole 3 in CDCl_3_, Figure S-11, LC-MS of Triazole 3, Figure S-12, ^1^H NMR of Triazole 4 in DMSO, Figure S-13, LC-MS characterization of Triazole 4, Figure S-14, ^1^H NMR of Triazole 5 in DMSO-d6, Figure S-15, LC-MS characterization of Triazole 5, Figure S-16, ^1^H-NMR of lyophilized CuAAC product Triazole 5 and starting material, Method S-3, Calculation of percentage yield of Triazole 5, Figure S-17, ^1^H-NMR of Triazole 6 mixture with 43% conversion, Figure S-18, LC-MS reaction mixture of Triazole 6 and remaining azide, Figure S-19, Purification of Triazole 7, Figure S-20, LC-MS of purified Triazole 7, Figure S-21, ^1^H-NMR of HPLC purified Triazole 7, Method S-4, Calculation of conversion to Triazole 7, Figure S-22, Determination of conversion to triazole 7 by ^1^H-NMR, Figure S-23, Monitoring of leaching of copper from resin using the absorbance of [Cu-(DDTC)_2_], List S-2, Cartesian coordinates of the optimized structures A, B, P, LnCu, I1, I1′, I2, TS1, I3, TS2, I4.
