# Supplementary material for: Development of Novel Immobilized Copper–Ligand Complex for Click Chemistry of Biomolecules
Source: Molecules. 2024 May 5;29(9):2148. doi: 10.3390/molecules29092148 (PMC11085236; doi:10.3390/molecules29092148)
Supplement: Supplementary file 1 [file molecules-29-02148-s001.zip › molecules-2957824-supplementary.pdf]

Supplementary Information

Development of novel immobilized copper-ligand complex for click chemistry of biomolecules

Rene Kandler[1], Yomal Benaragama[2], Dr. Manoranjan Bera[1], Caroline Wang[1], Rasheda Aktar Samiha[1], Dr. W. M. C. Sameera[2], Dr. Samir Das[1]*, Dr. Arundhati Nag[1]*

[1]Clark University

[2] University of Colombo

TABLE OF CONTENTS:

Figure S- 1: 1H NMR of N-(2-diphenylphosphaneyl)benzylidine)prop-2-yn-1-aminium 3
Figure S- 2: MS of reduced and purified ligand 4
Figure S- 3: Monitoring the formation of Complex 1 by FT-IR 5
Figure S- 4: ESI-MS of Complex **1**. 6
Figure S- 5: NMR of Complex **1** 7
List S- 1: Cartesian coordinates of the optimized structure of Complex **1** 9
Figure S- 6: Stability of Complex **1** in solution 11
Figure S- 7: LC-MS of Triazole 1 12
Method S- 1: Quantification of CuAAC reaction yield from NMR 13
Method S- 2: Monitoring of Triazole 1 formation in solutions with 50 % or more water content 15
Figure S- 8: ^{1}H NMR of Triazole 2 in $CDCl_3$. 16
Figure S- 9: LC-MS characterization of Triazole 2 17
Figure S- 10: ^{1}H NMR of Triazole 3 in $CDCl_3$. 18
Figure S- 11: LC-MS of Triazole 3 19
Figure S- 12: ^{1}H NMR of Triazole 4 in DMSO. 20
Figure S- 13: Lc-MS characterization of Triazole 4 21
Figure S- 14: ^{1}H NMR of Triazole 5 in DMSO-d_6 22
Figure S- 15: LC-MS characterization of Triazole 5. 23
Figure S- 16: ^{1}H-NMR of lyophilized CuAAC product Triazole 5 and starting material 24
Method S- 3: Calculation of percentage yield of Triazole 5 24
Figure S- 17: ^{1}H-NMR of Triazole 6 mixture with 43% conversion 25
Figure S- 18: LC-MS reaction mixture of Triazole 6 and remaining azide 26
Figure S- 19:Purification of Triazole 7 27
Figure S- 20: LC-MS of purified Triazole 7. 28
Figure S- 21: ^{1}H-NMR of HPLC purified Triazole 7 29
Method S- 4: Calculation of conversion to triazole 7 30
Figure S- 22: Determination of conversion to triazole 7 by ^{1}H-NMR 30
Figure S- 23: Monitoring of leaching of copper from resin using the absorbance of [Cu-$(DDTC)_2$] 32
List S- 2: Cartesian coordinates of the optimized structures A, B, P, LnCu, I1, I1’, I2, TS1, I3, TS2, I4........ 34

Figure S- 1: ^{1}H NMR of N-(2-diphenylphosphaneyl)benzylidine)prop-2-yn-1-aminium

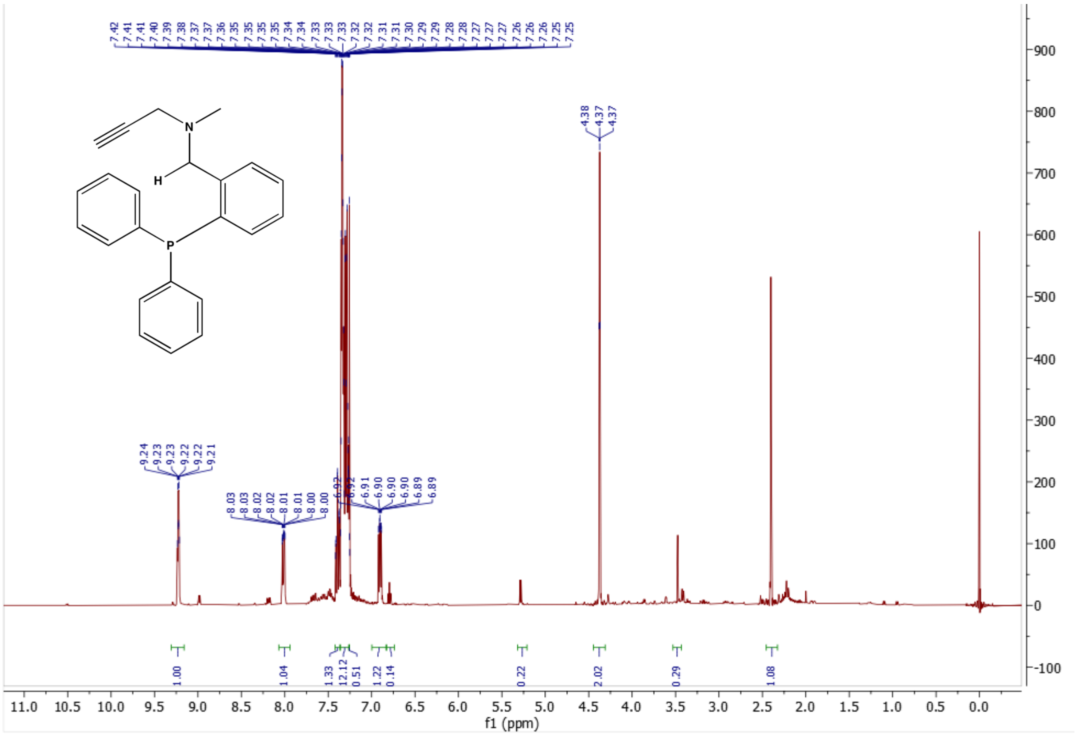

Figure S- 2: MS of reduced and purified ligand

Expected value for m/z: $(M+H)^+$ 344.2 detected 344.1. There is also an oxidized ligand present under LC-MS conditions $(M+O+H)^+$.

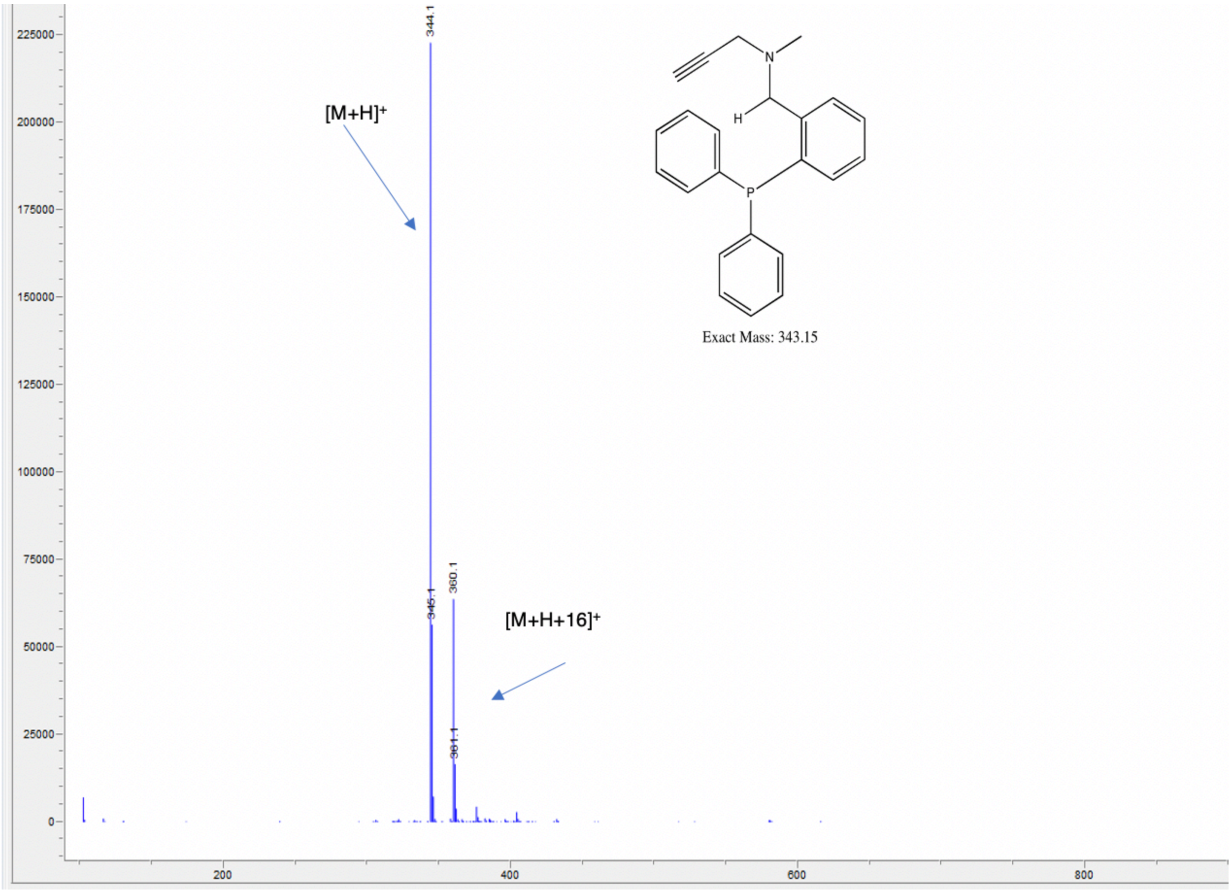

*Figure S- 3: Monitoring the formation of Complex **1** by FT-IR*

Monitoring the condensation to synthesize imine. A. incomplete conversion of aldehyde to imine after 24h; B. nearly completed conversion after 52 hours

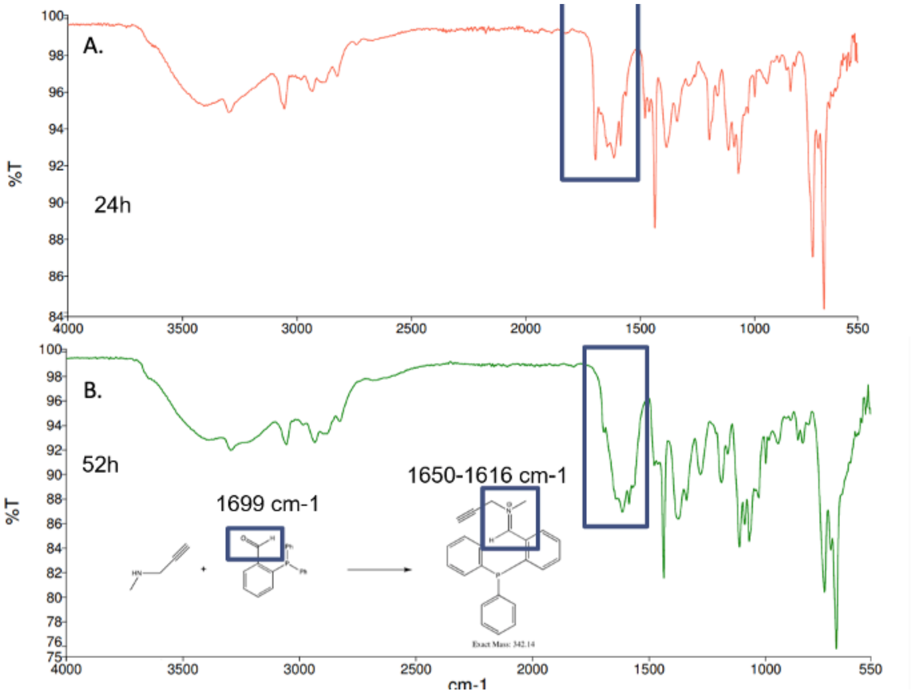

*Figure S- 4: ESI-MS of Complex **1**.*

Expected mass $[M]^+$ 521.1, found 521.1

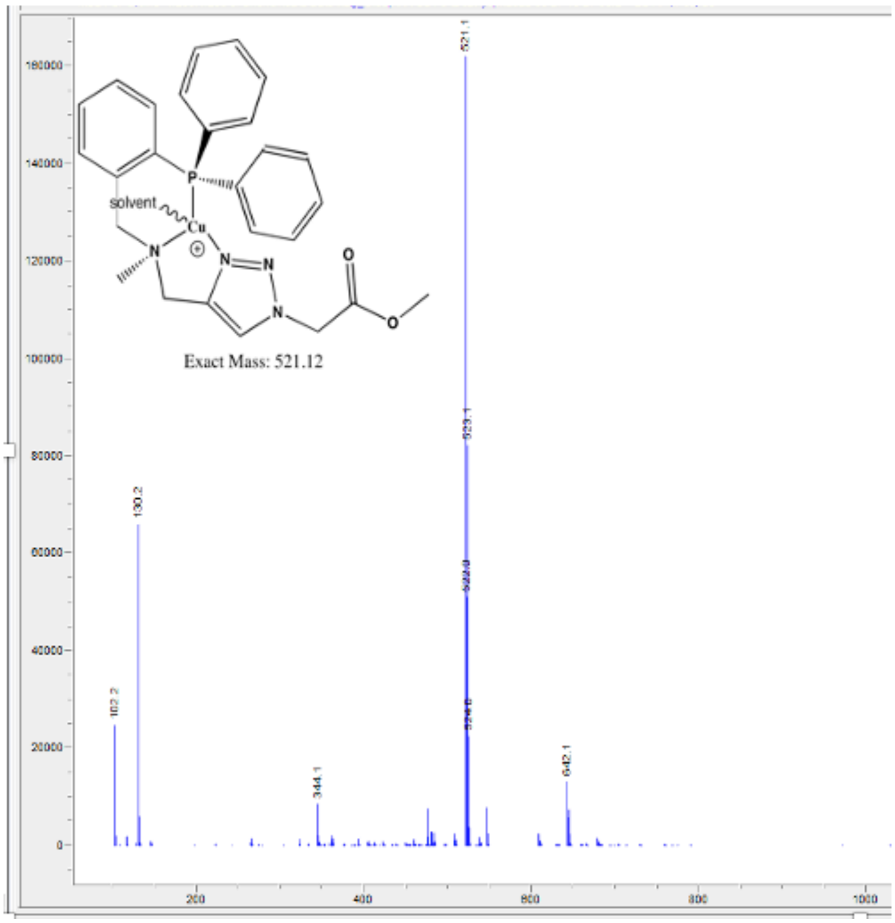

Figure S- 5: NMR of Complex ***1***

A. ^{1}H NMR of Complex **1**

^{1}H NMR (400 MHz, CD_3CN) δ 7.82 (s, 1H), 7.54 – 7.48 (m, 3H), 7.48 – 7.40 (m, 13H), 7.38 (dd, J = 7.5, 1.3 Hz, 1H), 7.36 – 7.30 (m, 4H), 6.94 (dd, J = 8.7, 7.5, 1.3 Hz, 1H), 5.26 (s, 2H), 3.95 (s, 1H), 3.89 (s, 3H), 3.76 (s, 1H), 3.46 (s, 2H), 2.47 (s, 3H).

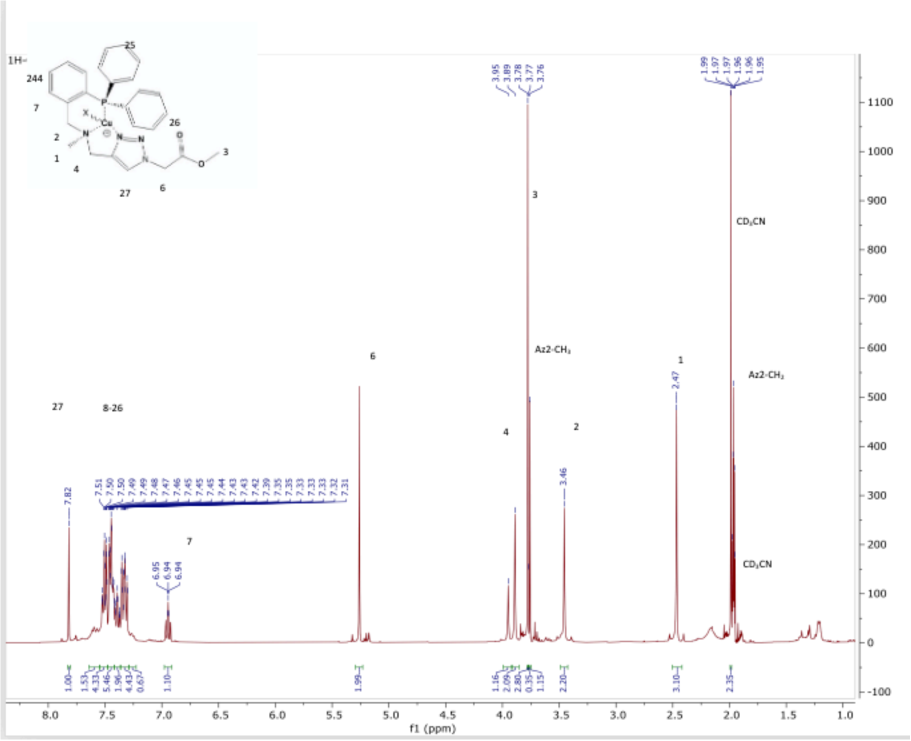

B. ^{13}C NMR of Complex **1**.

(101 MHz, CD_3CN) δ 166.83, 145.14, 139.90, 139.73, 134.04, 133.95, 133.72, 133.56, 133.12, 131.81, 131.53, 130.99, 130.57, 130.48, 129.22, 129.17, 129.13, 129.03, 124.15, 62.84, 62.77, 54.27, 52.67, 52.03, 51.29, 49.76, 44.24

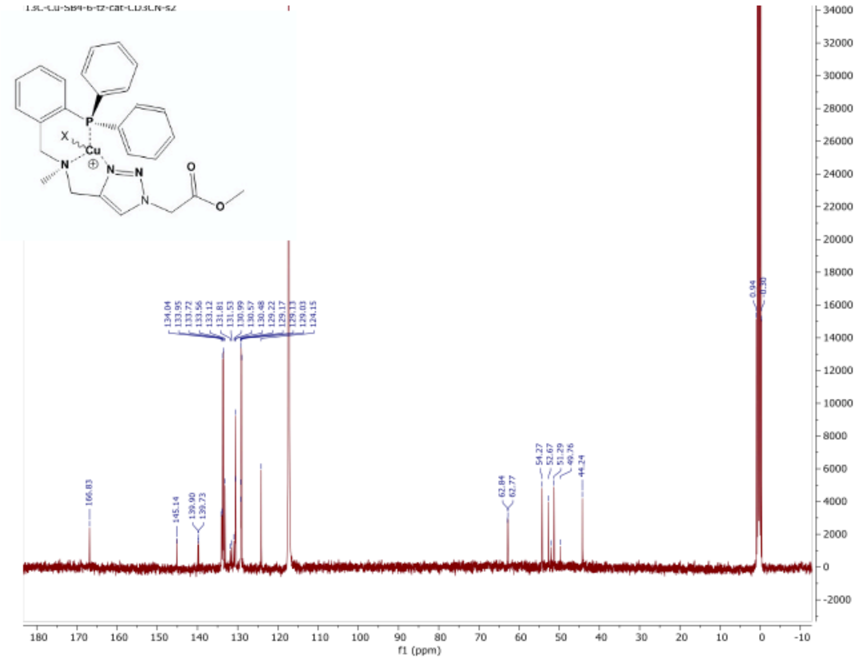

*List S- 1: Cartesian coordinates of the optimized structure of Complex **1***

C -1.15964100 0.50015000 2.92826400
H -1.50346000 1.47863900 2.56880000
H -1.13800700 0.55571600 4.03048200
N 0.16866600 0.27485300 2.36298000
Cu -0.49622200 1.72836300 -0.18886700
C 0.79305700 -0.96143000 2.82306000
H 1.01557900 -0.93667100 3.90458900
H 1.72595400 -1.12004200 2.27476800
C 1.06527800 1.42265300 2.55150100
H 1.62839000 1.36083000 3.49515100
H 0.46011800 2.33382400 2.59997300
C 2.00564400 1.53892300 1.39427300
C 3.36823200 1.40000700 1.27304700
H 4.14419200 1.18755900 1.99062600
C 4.88322400 1.47364600 -0.75663800
H 5.62929700 2.10259100 -0.26385900
H 4.72717100 1.85923800 -1.76488300
C 5.44887000 0.05913700 -0.85627200
C 5.25883700 -2.18313300 -0.15794600
H 4.53678200 -2.76246000 0.41329900
N 1.51940500 1.79283900 0.14207600
N 3.62000100 1.57756800 -0.05009700
N 2.49073200 1.81198800 -0.73570000
H 0.13487100 -1.80730000 2.62046700
H 6.24362600 -2.20887900 0.31279600
H 5.32848700 -2.55424300 -1.18196700
O 6.44681500 -0.17918900 -1.50120300
O 4.74951000 -0.83122700 -0.15302500
P -1.34742300 -0.32686100 -0.22473100
C -0.05696600 -1.60815000 -0.45106900
C -0.15546400 -2.89357500 0.09758200
C 1.05199800 -1.28548300 -1.24742300
C 0.84403400 -3.83857400 -0.14145600
H -1.00978500 -3.15880000 0.71110700
C 2.04342600 -2.23386600 -1.49398700
H 1.14270500 -0.29186100 -1.67387600
C 1.94326700 -3.51199600 -0.93826200
H 0.76029500 -4.83059300 0.29240600
H 2.89427600 -1.97156100 -2.11451500
H 2.71709700 -4.25015400 -1.12704000
C -2.43708400 -0.56728900 -1.68581200
C -3.36611800 0.44570600 -1.97397700
C -2.36945800 -1.69185900 -2.51965200
C -4.22296700 0.32828200 -3.06738000
H -3.41401100 1.32782900 -1.34059700

C -3.22233100 -1.80285000 -3.62012100
H -1.65453300 -2.48037000 -2.30965100
C -4.15069900 -0.79662000 -3.89411200
H -4.93988800 1.11616700 -3.27899200
H -3.16147400 -2.67733800 -4.26134000
H -4.81242000 -0.88531300 -4.75061000
C -2.37640000 -0.95001000 1.17452000
C -2.16739900 -0.55273500 2.51572700
C -3.38317300 -1.88648500 0.89009200
C -2.96178000 -1.12231700 3.51698000
C -4.17043200 -2.43690500 1.90095000
H -3.55442000 -2.19245000 -0.13579800
C -3.95711800 -2.05412500 3.22328500
H -2.79355500 -0.82435300 4.54812300
H -4.93951700 -3.16162600 1.65179200
H -4.55685700 -2.47730500 4.02341100
N -1.38903800 3.45856800 -0.32687800
C -1.97127900 4.45315200 -0.43309100
C -2.70560900 5.70099200 -0.56788000
H -3.27806000 5.89035800 0.34478900
H -2.00807900 6.52684200 -0.73388200
H -3.39363800 5.63324100 -1.41554300

*Figure S- 6: Stability of Complex **1** in solution.*

The stability of Complex **1** in CD_3CN was monitored by ^{1}H-NMR for seven days. A) dissolved catalyst after a few hours; B) after 4 days; C) after 6 days; D) after 7 days in solution.

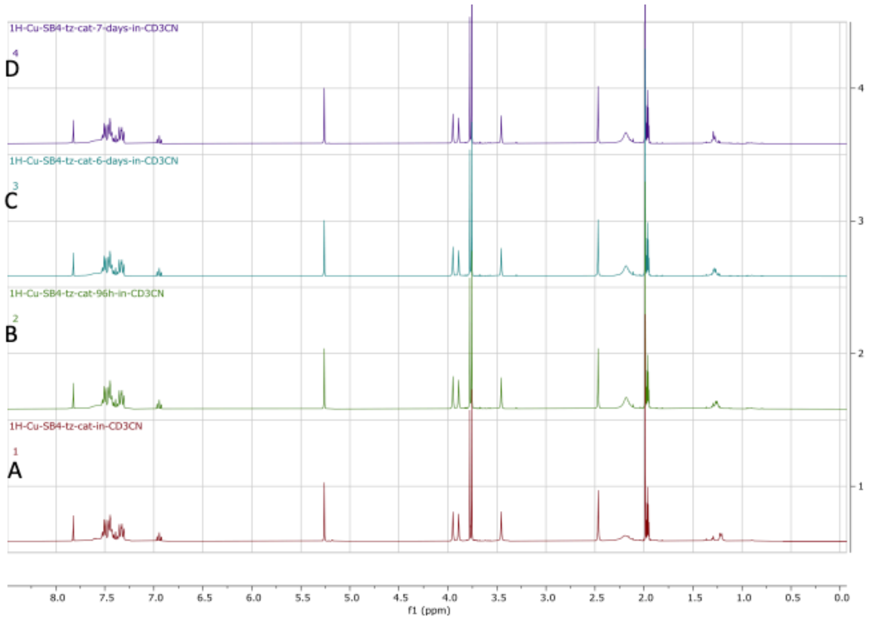

Figure S- 7: LC-MS of Triazole 1

The LC of the click reaction showed a peak at around 4 minutes, which showed the expected mass of Triazole 1 (Exact Mass: 235.11, observed m/z 236.1, $[M+H]^+$)

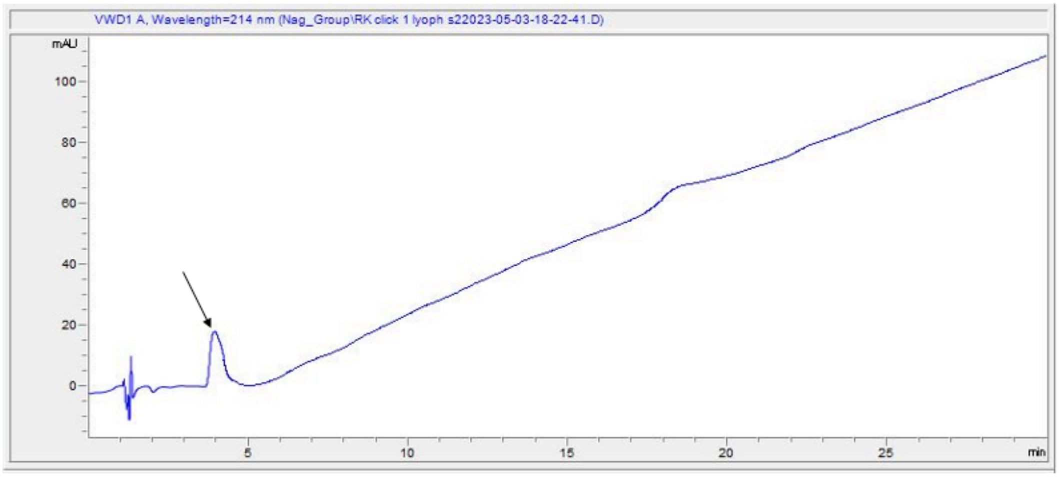

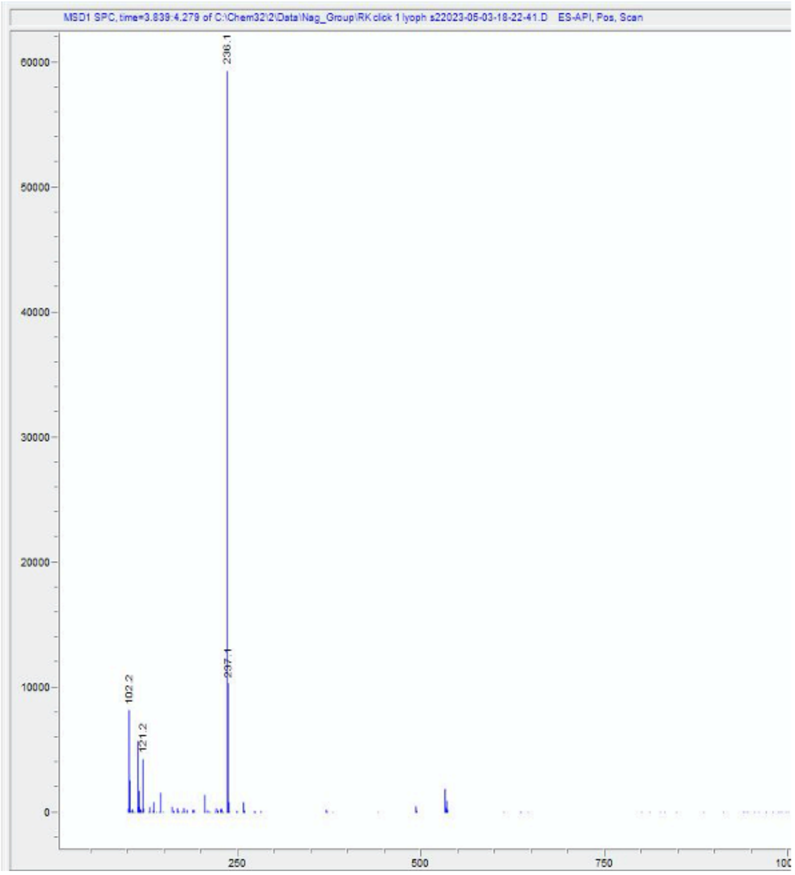

Method S- 1: Quantification of CuAAC reaction yield from NMR

Quantification of CuAAC reactions that precipitate by dissolving lyophilized reaction mixture with a high percentage of acetonitrile (80 or higher) and calculating the percentage yield from the NMR of this solution: When CuAAC reactions precipitated with higher triazole formation, the reaction was stopped, the reaction mixture was lyophilized and then dissolved in a solvent with 80% or more acetonitrile, and the yield of the triazole was calculated from the NMR of the solutions with high percentages of acetonitrile.

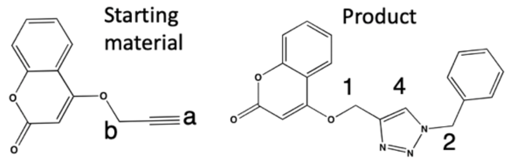

The conversion was calculated from every measured sample by integrating the proton at position a of the starting material (terminal alkyne) and normalizing that integral to be one. The peaks of each proton at positions 1, 2, and 4 of the Triazole product are integrated, and each percentage yield is calculated with respect to the proton at position a. A similar calculation was done normalizing the integral for protons at position b of the starting material (CH_2 azide) to 2 and then integrating peaks at positions 1, 2 or 4 to and calculating the % yield for each position with respect to proton b. The average percentage yield value was calculated by averaging the percentage yields for position 1 with respect to (wrt) position a, position 1 wrt position b, position 4 wrt position a, position 4 wrt position b, position 2 wrt position a, and position 2 wrt position b (therefore an average of six percentage yield calculations). Table Con1 show the normalization calculations in which the integral at position 4 is normalized to 1. Table Con2 lists the % conversation calculations for position 1 wrt position a, position 1 wrt position b, etc, which were then averaged to give the final average yield of a reaction cycle. The yields for 3 reaction cycles are shown in Table Con2.

Table Con1

Reaction	time (h)	Position 1 (2 protons)	Position 2 (2 protons)	Position 4 (1 proton)	Position a (1 proton)	Position b (2 protons)
click 31 lyoph cycle 1	24	2.07	2.06	1	0.93	1.94
click 33 lyoph cycle 2	24	2.02	2.09	1	0.41	0.85
click 34 lyoph cycle 3	24	1.96	1.98	1	0.29	0.6

Table Con2

Reaction	time (h)	1:b	2:b	4:b	1:a	2:a	4:a	Yield triazole Average*
click 31 lyoph cycle 1	24	$\frac{\left(\frac{2.07}{2}\right)*100}{[(\frac{2.07}{2}+\frac{1.94}{2})]}=$ 51.6	51.5	50.8	$\frac{\left(\frac{2.07}{2}\right)*100}{[(\frac{2.07}{2}+0.93)]}=$ 52.7	52.6	51.8	51.8
click 33 lyoph cycle 2	24	70.4	71.1	70.2	71.1	71.8	70.9	70.9
click 34 lyoph cycle 3	24	76.6	76.7	76.9	77.2	77.3	77.5	77.0

Method S- 2: Monitoring of triazole 1 formation in solutions with 50 % or more water content

TMS was used as a standard for comparison of CuAAC reactions done in solvents with 50% water or more. In the closed tube, the amount of TMS was assumed to be constant. The TMS integral was set to 1.0, and the percentage yield was calculated by subtracting the changed integral for the alkyne proton from the integral value for the alkyne proton at 0 hours, and calculating the change as a percentage of the integral of alkyne proton 1 at 0 hours.

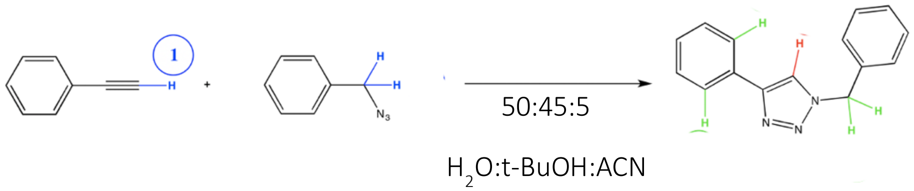

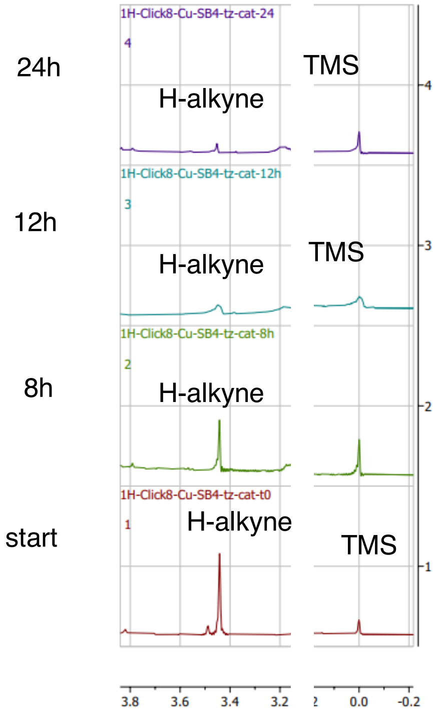

time (h)	alkyne term H	TMS	conversion by H-Alkyne : TMS
0	8.76	1	0.0
2	3.76	1	57.1
4	2.82	1	67.8
8	2.16	1	75.3
12	0.17	1	98.1
16	0.28	1	96.8
20	0.30	1	96.6
24	0.30	1	96.6
28	0.37	1	95.8
28**	0.37	1	95.8

Figure S- 8: ^{1}H NMR of Triazole 2 in $CDCl_3$.

Triazole 2: 1-benzyl-4-(phenoxymethyl)-1*H*-1,2,3-triazole

^{1}H NMR (400 MHz, $CDCl_3$) δ 7.53 (s, 1H), 7.41 – 7.33 (m, 3H), 7.32 – 7.23 (m, 5H), 7.01 – 6.92 (m, 3H), 5.52 (s, 2H), 5.18 (s, 2H).

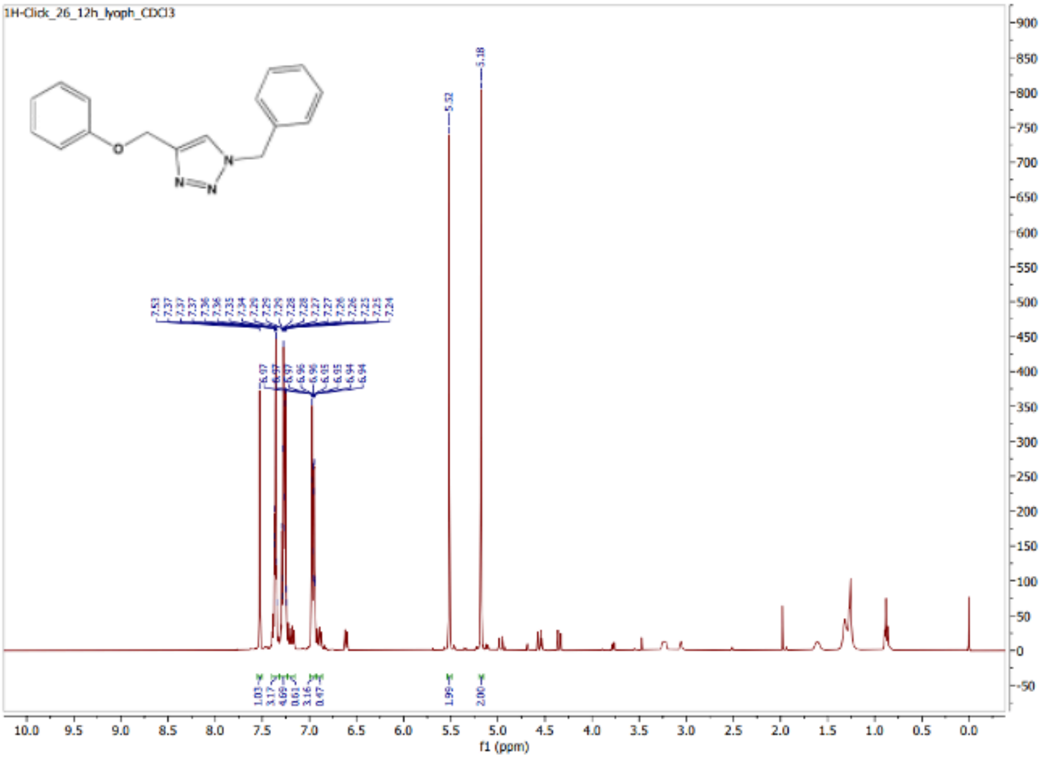

Figure S- 9: LC-MS characterization of Triazole 2

LC trace shows a peak at 5 minutes, which, when analyzed in the MS, shows $(M+H)^+$ value of 267.1 (Expected exact mass of M: 266.12).

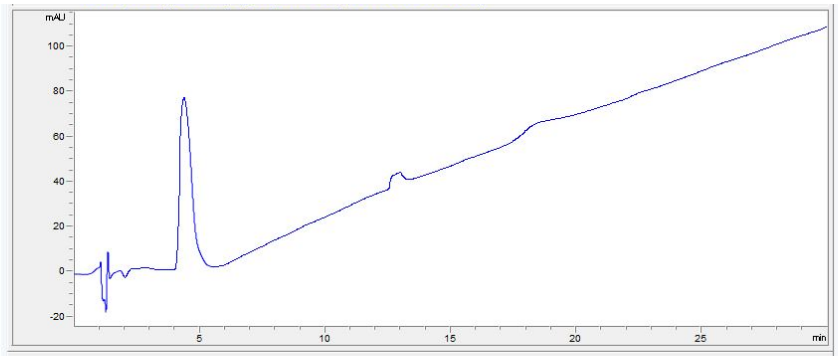

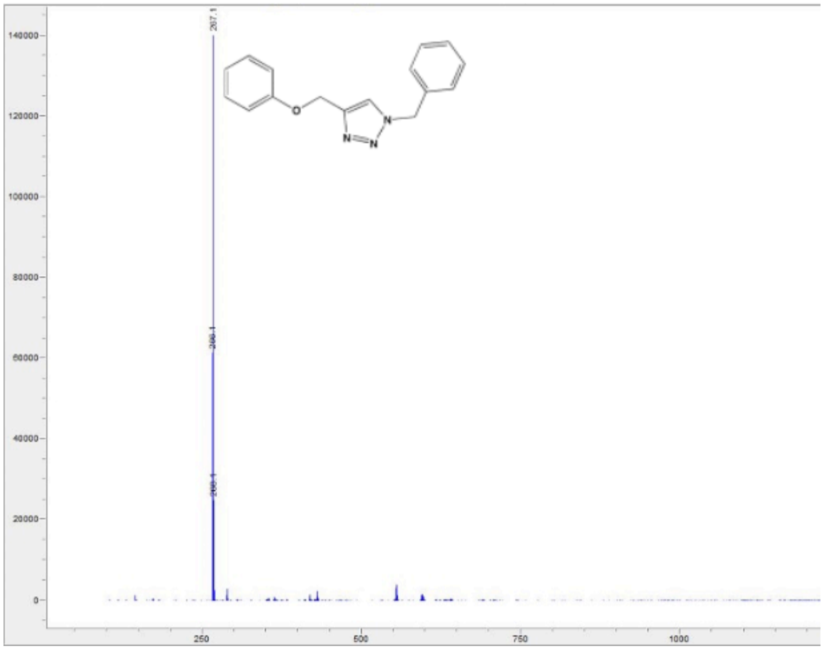

Figure S- 10: ^{1}H NMR of Triazole 3 in $CDCl_3$.

^{1}H NMR (400 MHz, $CDCl_3$) δ 7.76 (dt, J = 8.4, 2.6 Hz, 1H), 7.63 (s, 1H), 7.54 (ddd, J = 8.7, 7.3, 1.7 Hz, 1H), 7.45 – 7.35 (m, 3H), 7.31 (ddd, J = 8.4, 4.3, 1.8 Hz, 3H), 7.25 – 7.19 (m, 1H), 5.84 (s, 1H), 5.58 (s, 2H), 5.31 (s, 2H).

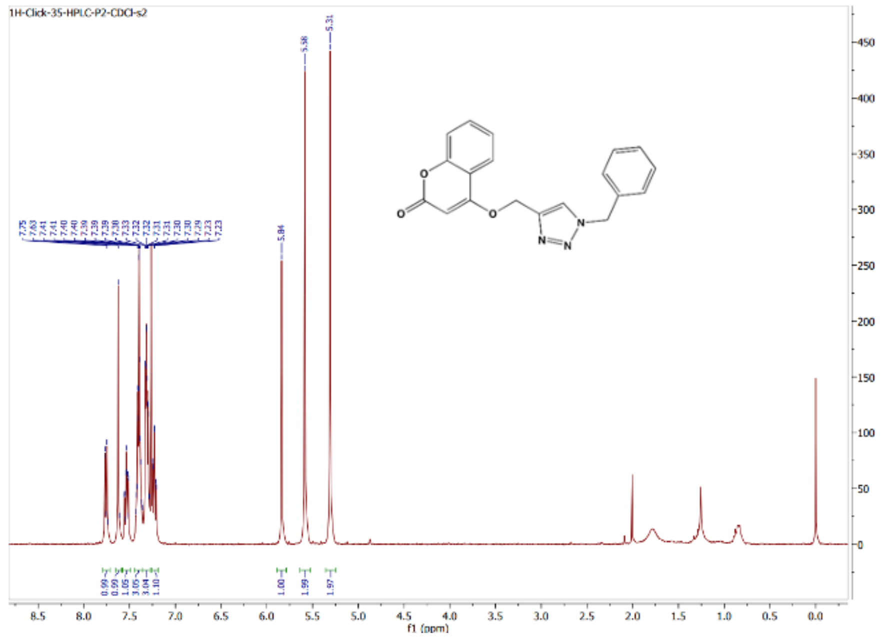

Figure S- 11: LC-MS of Triazole 3

LC of triazole 3 shows a peak at 3 minutes, which is analyzed in MS. m/z values corresponding to $(M+H)^+$ (expected 334.1, observed 334.0;) $(M+Na)^+$ (expected 356.1, observed 356.0) and $(2M+H)^+$ (expected 667.3, observed 667.2

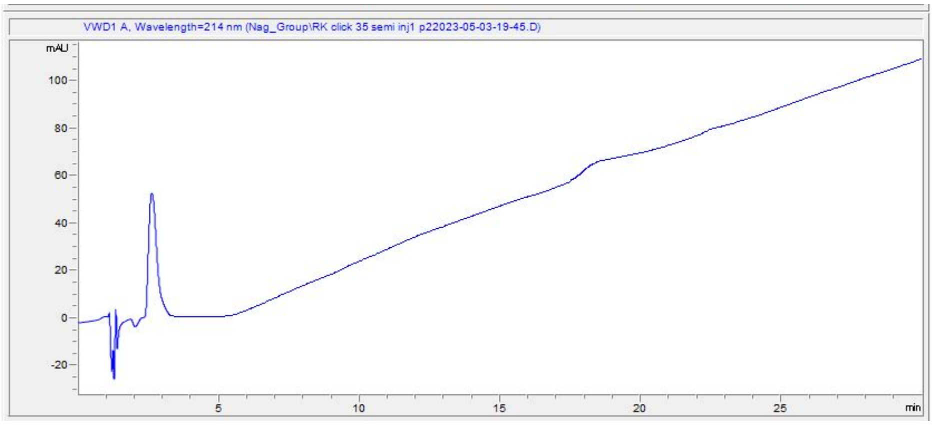

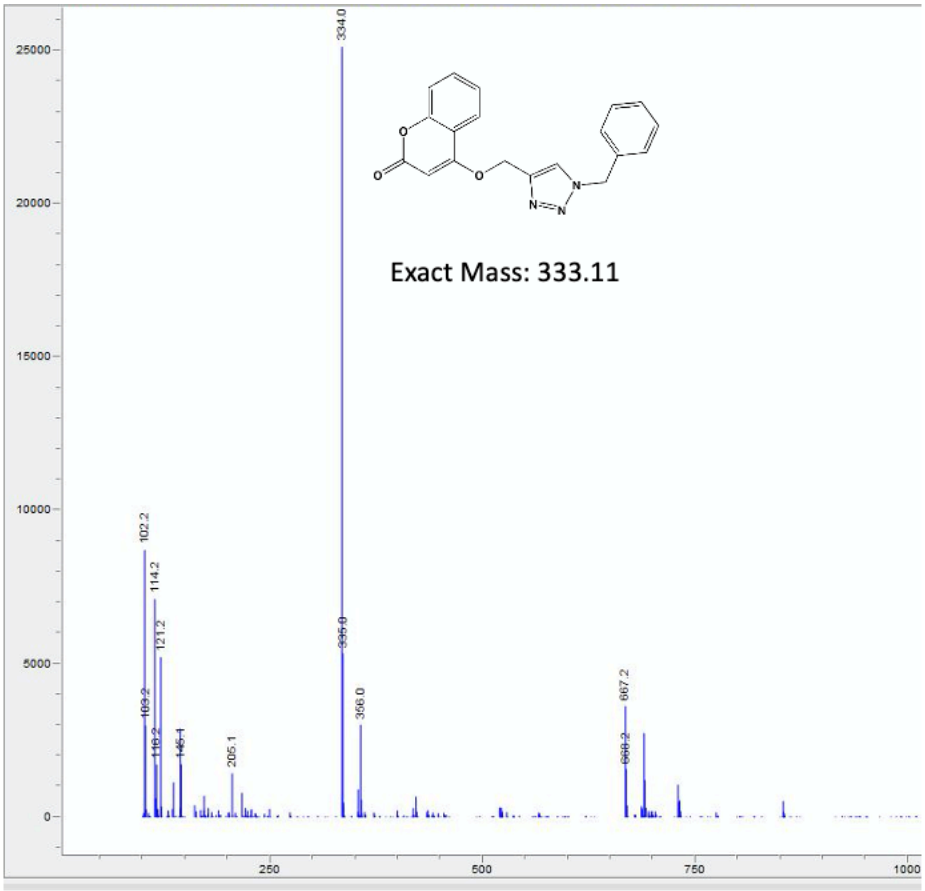

Figure S- 12: ^{1}H NMR of Triazole 4 in DMSO.

^{1}H NMR (400 MHz, dmso) δ 7.48 – 7.30 (m, 5H), 5.66 (d, J = 9.9 Hz, 2H), 0.30 (d, J = 1.1 Hz, 6H). Proton of triazole ring (CH) no signal due to D-H exchange, In DMSO lower CH_3 proton count (6 instead of 9).

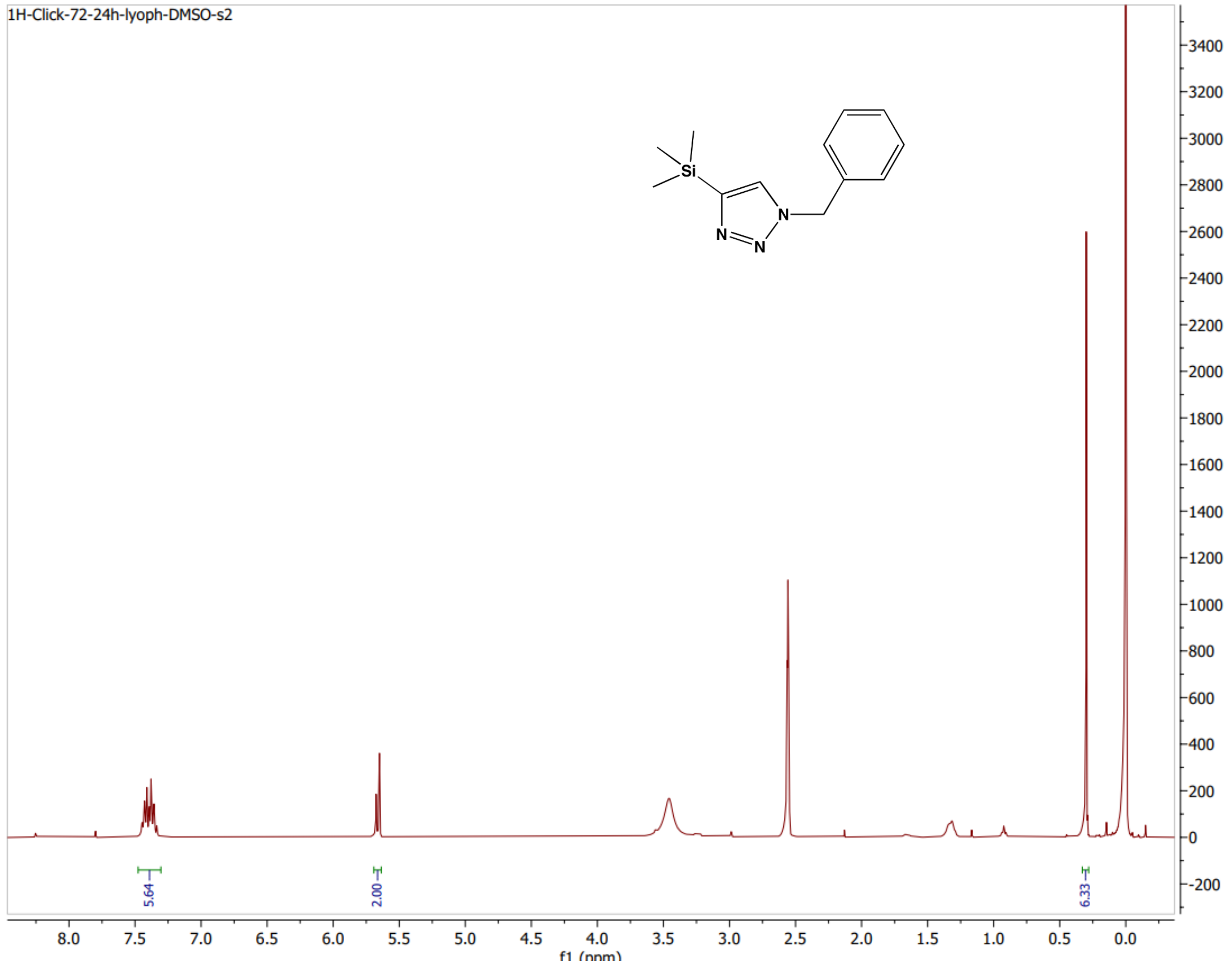

Figure S- 13: LC-MS characterization of Triazole 4.

LC of Triazole 4 shows a peak at 5 minutes, which is analyzed in MS. m/z values corresponding to $(M+D)^+$ (expected 233.1, observed 233.1) is observed.

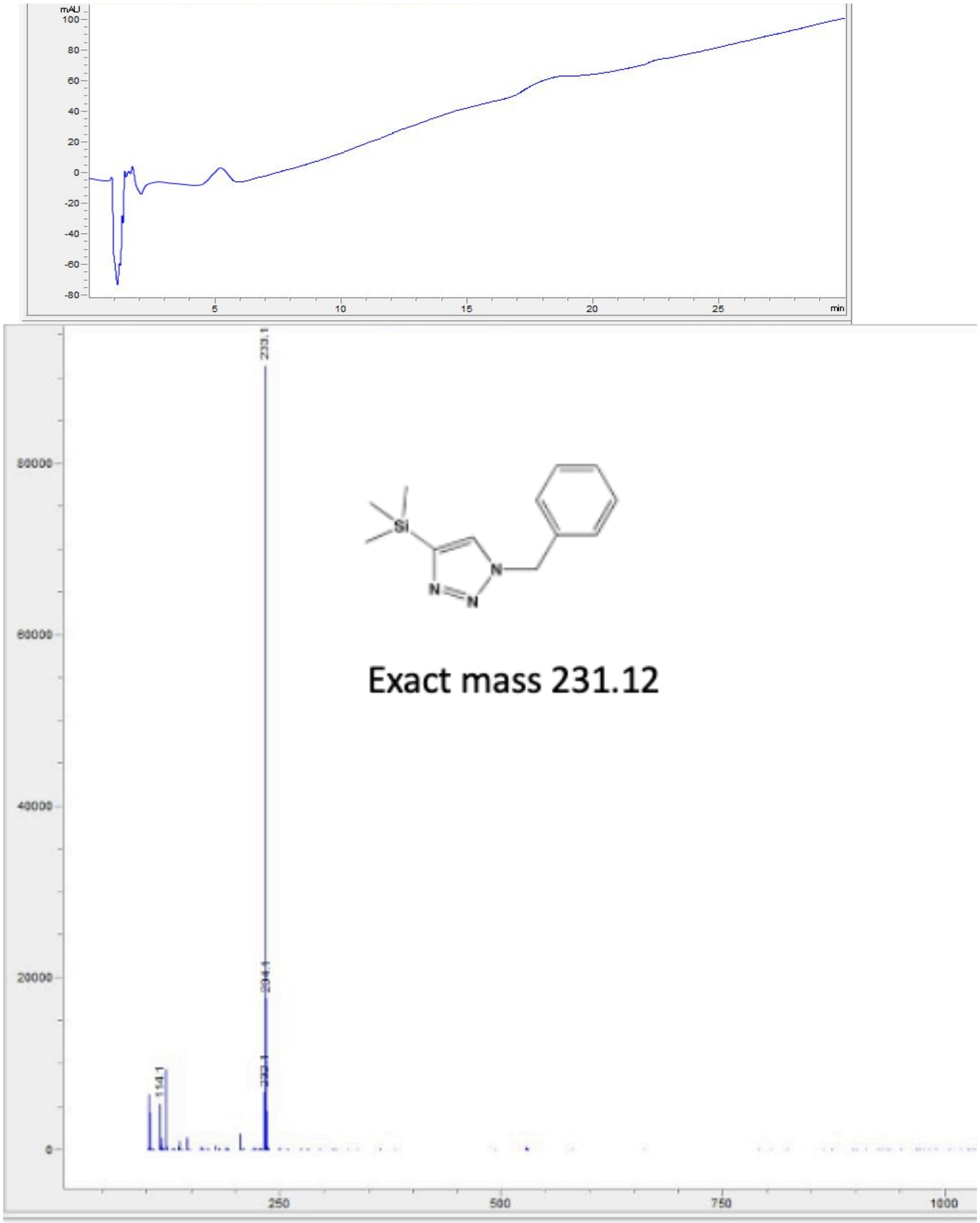

Figure S- 14: ^{1}H NMR of Triazole 5 in DMSO-d_6

^{1}H NMR (400 MHz, DMSO-d_6) δ 10.68 (s, 1H), 8.67 (d, J = 3.6 Hz, 1H), 7.84 (d, J = 7.4 Hz, 2H), 7.66 (d, J = 8.6 Hz, 1H), 7.56 – 7.39 (m, 2H), 7.32 (t, J = 7.6 Hz, 1H), 6.81 (d, J = 8.8 Hz, 1H), 6.75 (s, 1H), 5.91 (s, J = 7.0 Hz, 2H), 5.75 (s, J = 5.7 Hz, 1H).

8.67 ppm lower signal by D-H exchange is observed due to the presence of D_2O during click reaction.

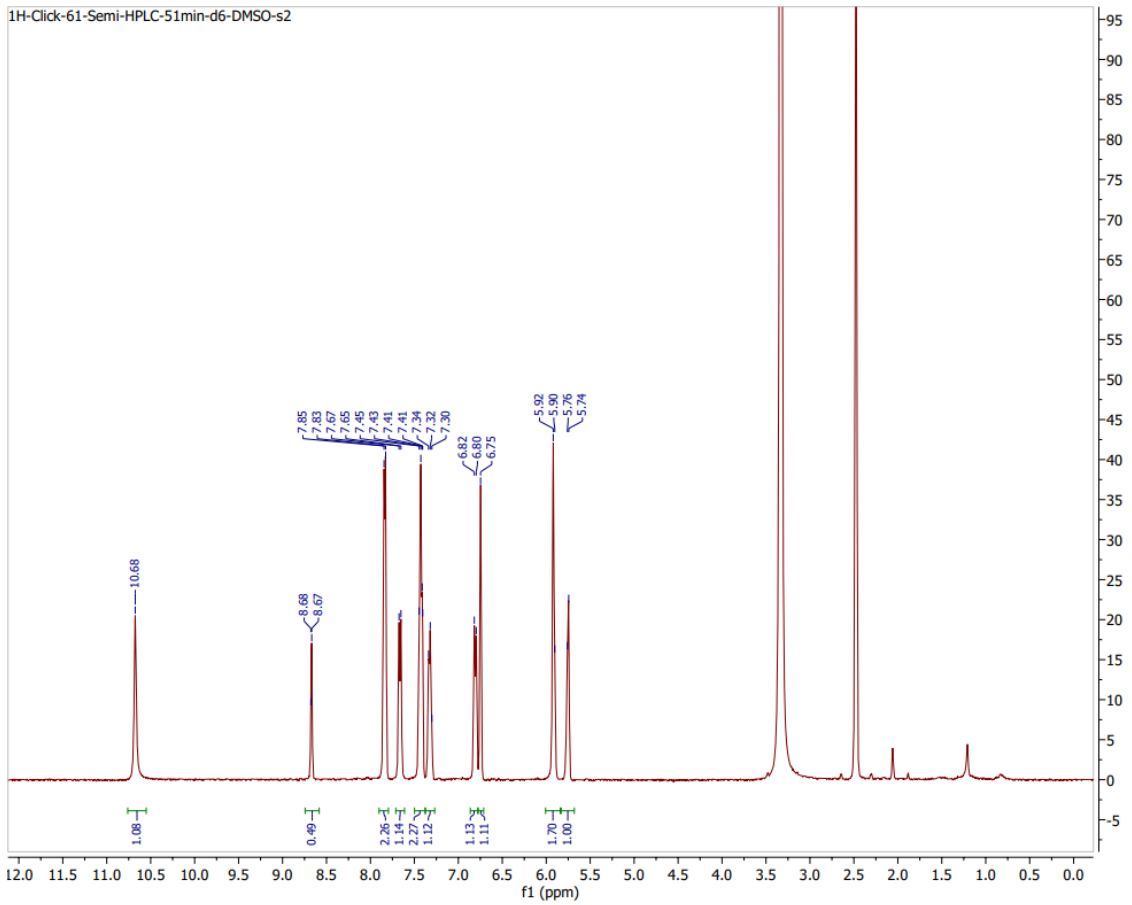

Figure S- 15: LC-MS characterization of Triazole 5.

LC of Triazole 5 shows a peak at 2 minutes, which is analyzed in MS. m/z values corresponding to $(M+D)^{+}$ (expected 319.1, observed 321.1) is observed.

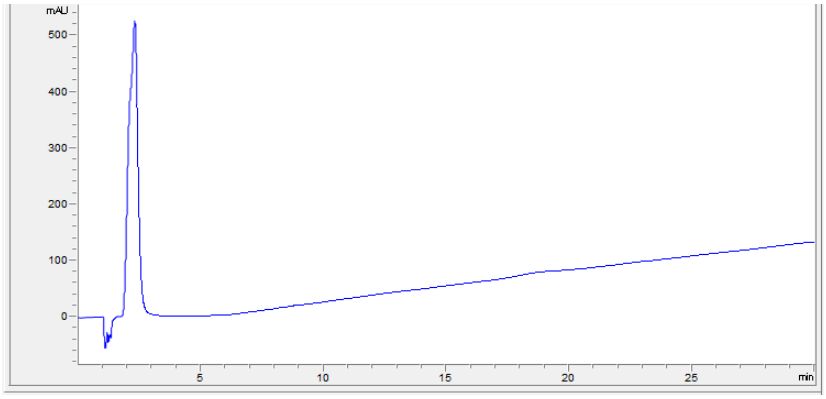

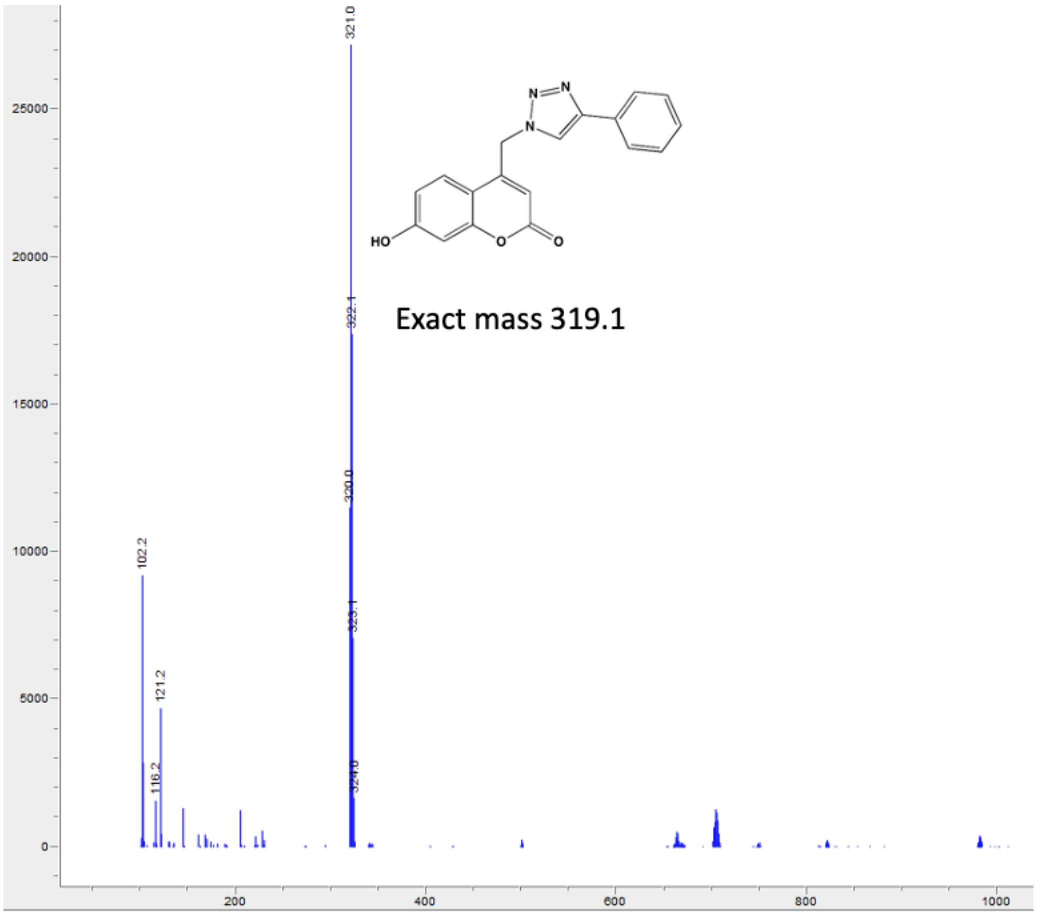

Method S- 3: Calculation of percentage yield of Triazole 5.

The identification of the product portion shifts in the ^{1}H NMR was vital for the calculation of the conversion to triazole 5 (shown in Figure S-16) In Figure S-16C the ^{1}H NMR of 4-(azidomethyl)-7-hydroxy-2*H*-chromen-2-one shows the shifts of the starting material (azide). The spectrum of the sample in Figure S-17B showed a very small conversion to Triazole 5 (7-hydroxy-4-((4-phenyl-1*H*-1,2,3-triazol-1-yl)methyl)-2*H*-chromen-2-one) (red arrows). The conversion was high for the sample depicted in Figure S-16A and matched the chemical shifts indicated by the arrows in Figure S-16B. After lyophilization of the sample, unreacted azide and formed triazole remained in the sample. The triazole peaks that newly formed were integrated, normalized to one or two protons, and compared to the integral of the remaining azide-CH_2 shift at 4.75 ppm.

Figure S- 16: ^{1}H-NMR of lyophilized CuAAC product Triazole 5 and starting material.

A) high conversion to desired product (Triazole 5); B) very low conversion to Triazole 5 and remaining 4-(azidomethyl)-7-hydroxy-2H-chromen-2-one. The arrows indicate triazole based proton signals; C) starting material 4-(azidomethyl)-7-hydroxy-2H-chromen-2-one.

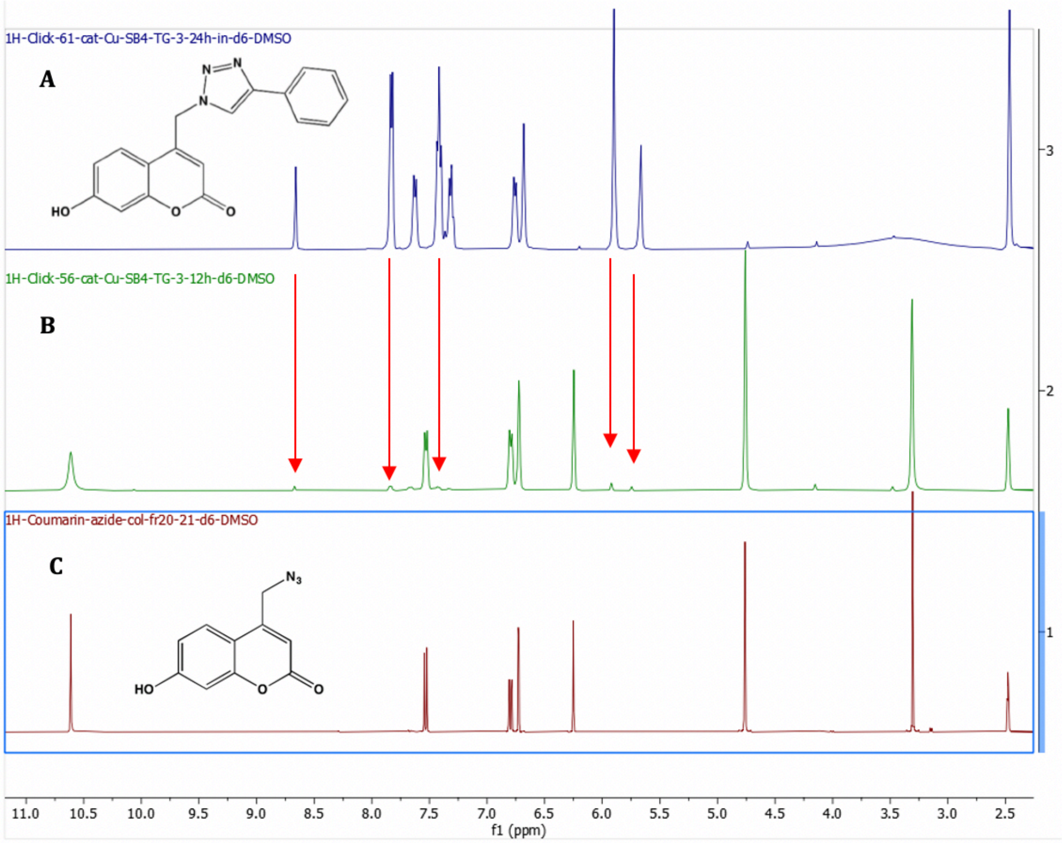

Figure S- 17: ¹H-NMR of Triazole 6 mixture with 43% conversion.

^{1}H NMR (400 MHz, DMSO-d6) δ 10.73 (s, 1H), 7.67 – 7.65 (d, 1H), 6.86 (s, 1H), 6.79 (d, J = 8.7 Hz, 1H), 5.89 (s, 1H), 5.64 (s, 1H), 4.76 (d, J = 11.6 Hz, 2H), 4.31 (s, 2H), 4.23 (d, J = 4.3 Hz, 2H), 4.01 (s, 5H).

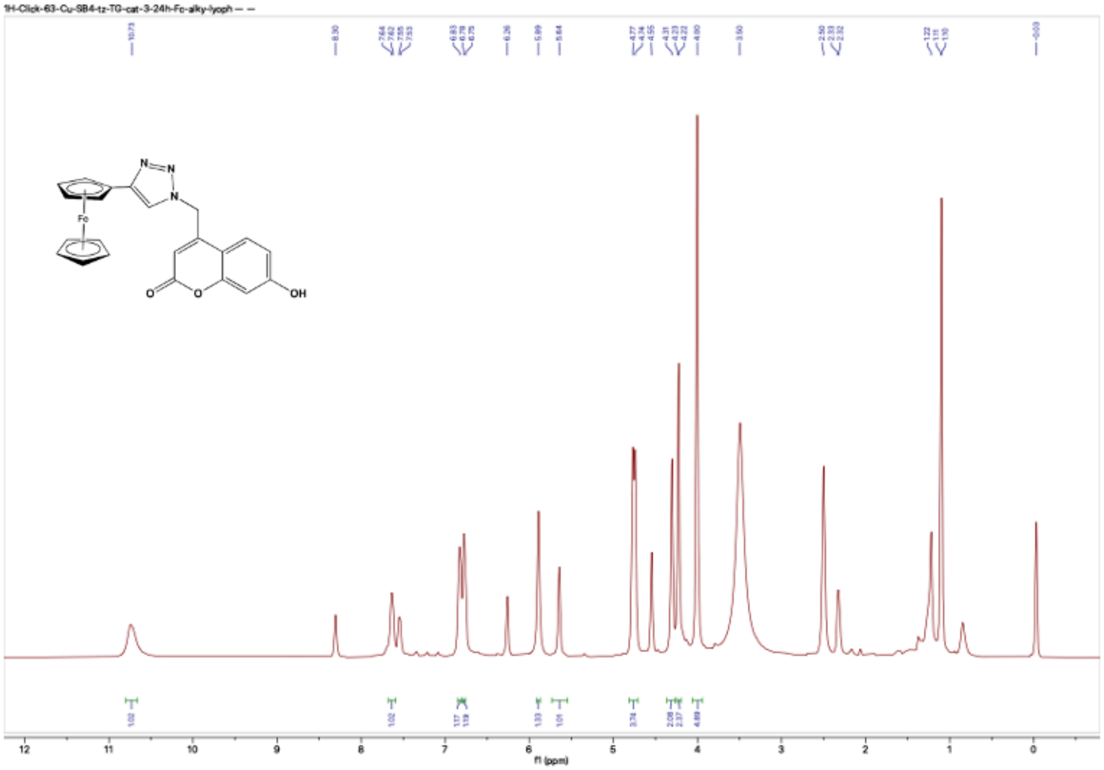

Figure S-18: LC-MS reaction mixture of Triazole 6 and remaining azide.

A. LC trace shows the azide and triazole, respectively. B. MS of Triazole 6 at 3.3 minutes shows m/z corresponding to $(M + \underline{D})^+$ (expected 429.0, observed 429.1).

A.

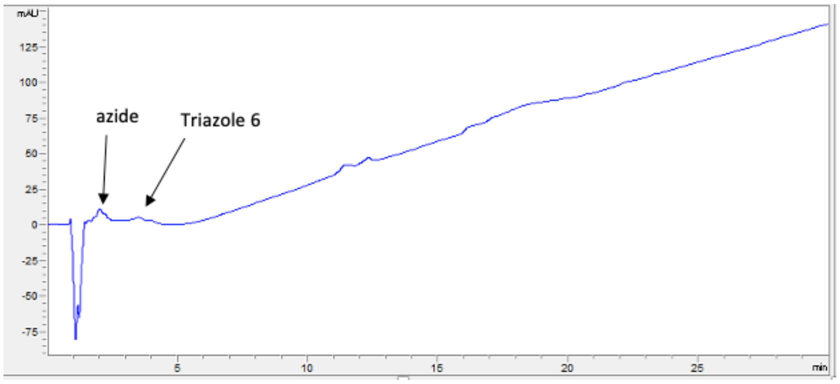

B.

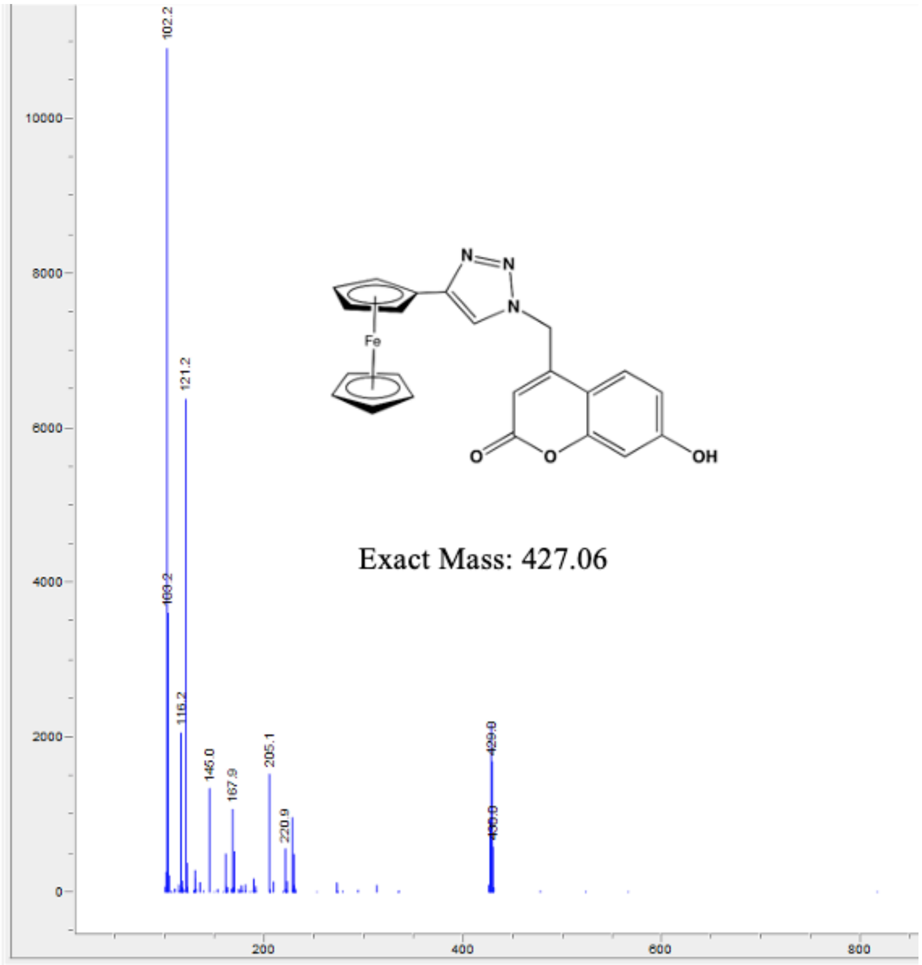

Figure S- 19: Purification of Triazole 7.

Part of the crude compound mixture, dissolved in DMSO, was analyzed and purified by reversed phase high performance liquid chromatography (RP-HPLC). The collected fractions P1, P2 (retention times 10.7 minutes, 11.2 minutes) had the same mass values. Using this method, a separation of the two triazole products was not possible (gradient pH 2 1% ACN – 90 % ACN, 70 min). The mixture of the triazole products was labeled Triazole 7.

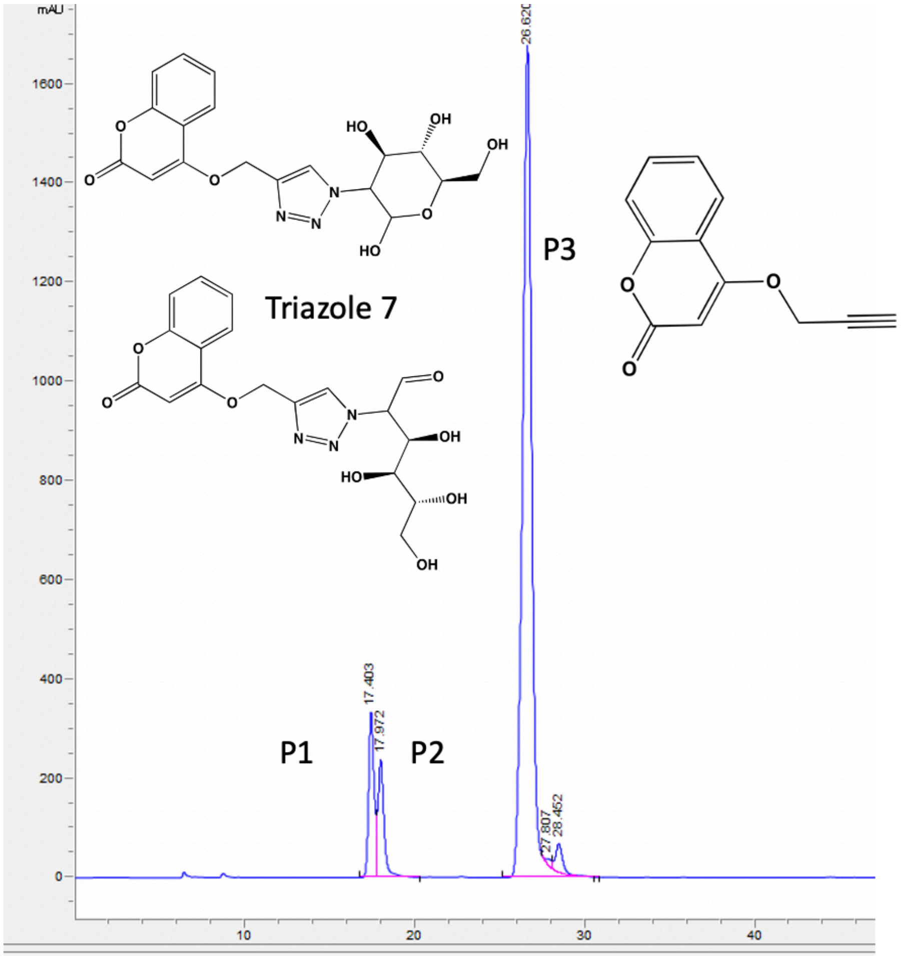

P1 and P2 had the same mass and was identified as the product Triazole 7. P3 was the starting material coumarin-alkyne.

Figure S- 20: LC-MS of purified Triazole 7.

LC of purified Triazole 7 shows a shoulder and a peak at 1 minute. MS shows m/z corresponding to [M + D]$^+$ (calculated 407.1, found 407.0) [M_{Deut} + Na]$^+$ (with 2 deuterium exchanges) calculated 429.1, found 429.0) and [2M + H]$^+$ (calculated 813.2, found 813.2).

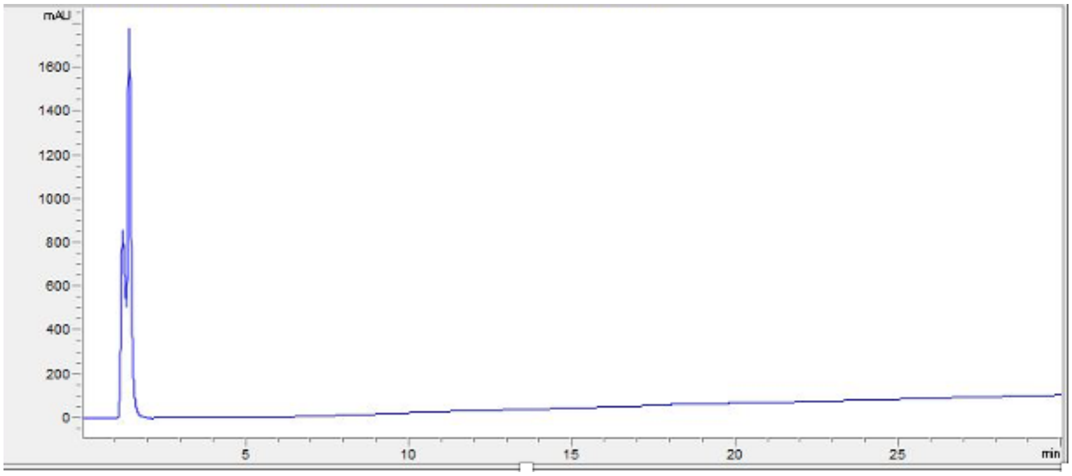

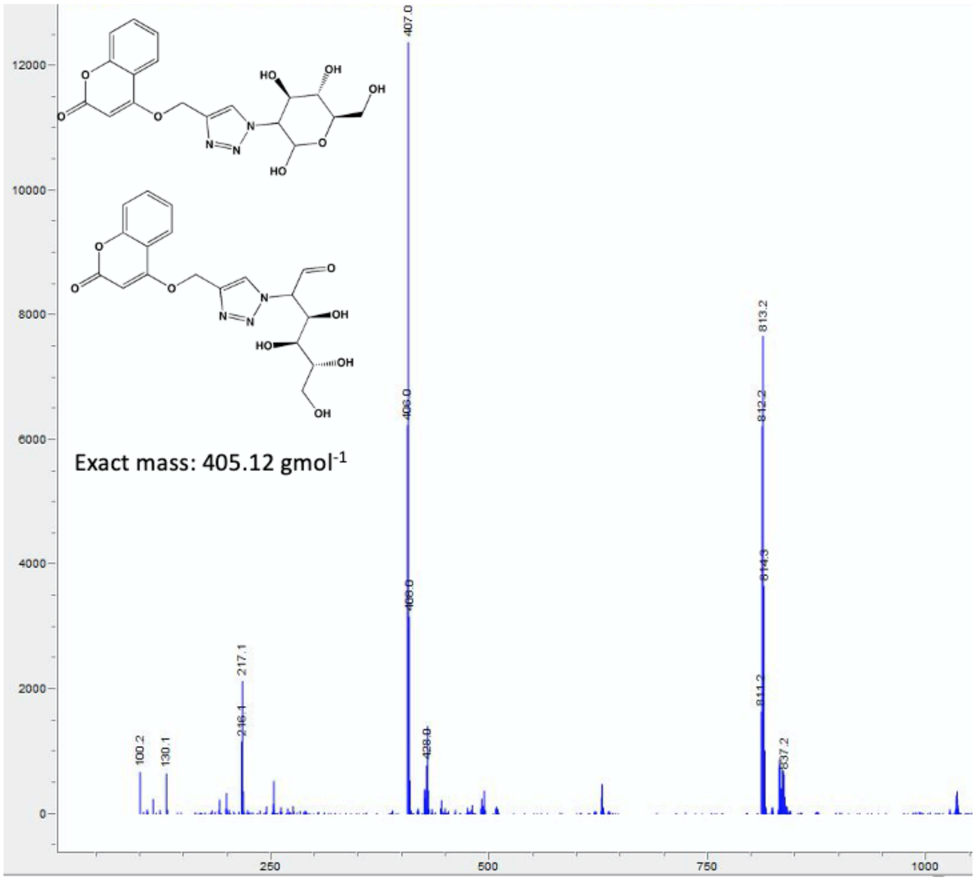

Figure S- 21: ^{1}H-NMR of HPLC purified Triazole 7.

^{1}H NMR (400 MHz, DMSO-d_6) δ 8.38 (s, 1H), 8.29 (s, 1H), 7.73 (ddd, *J* = 8.0, 6.4, 1.6 Hz, 1H), 7.64 (ddt, *J* = 8.7, 7.3, 1.5 Hz, 1H), 7.39 (dd, *J* = 8.4, 1.2 Hz, 1H), 7.33 (tt, *J* = 7.7, 1.6 Hz, 1H), 6.18 (d, *J* = 3.9 Hz, 1H), 5.40 (d, *J* = 3.1 Hz, 2H), 5.17 (d, *J* = 3.4 Hz, 1H), 4.97 (d, *J* = 8.2 Hz, 1H), 4.50 (dd, *J* = 10.9, 3.4 Hz, 1H), 4.13 – 4.02 (m, 1H), 3.92 (dd, *J* = 10.6, 8.5 Hz, 1H), 3.73 (dt, *J* = 11.7, 2.4 Hz, 1H), 3.67 (dd, *J* = 11.8, 2.2 Hz, 1H), 3.52 (ddd, *J* = 15.6, 11.8, 5.6 Hz, 2H), 3.38 – 3.29 (m, 1H), 3.27 – 3.19 (m, 1H).

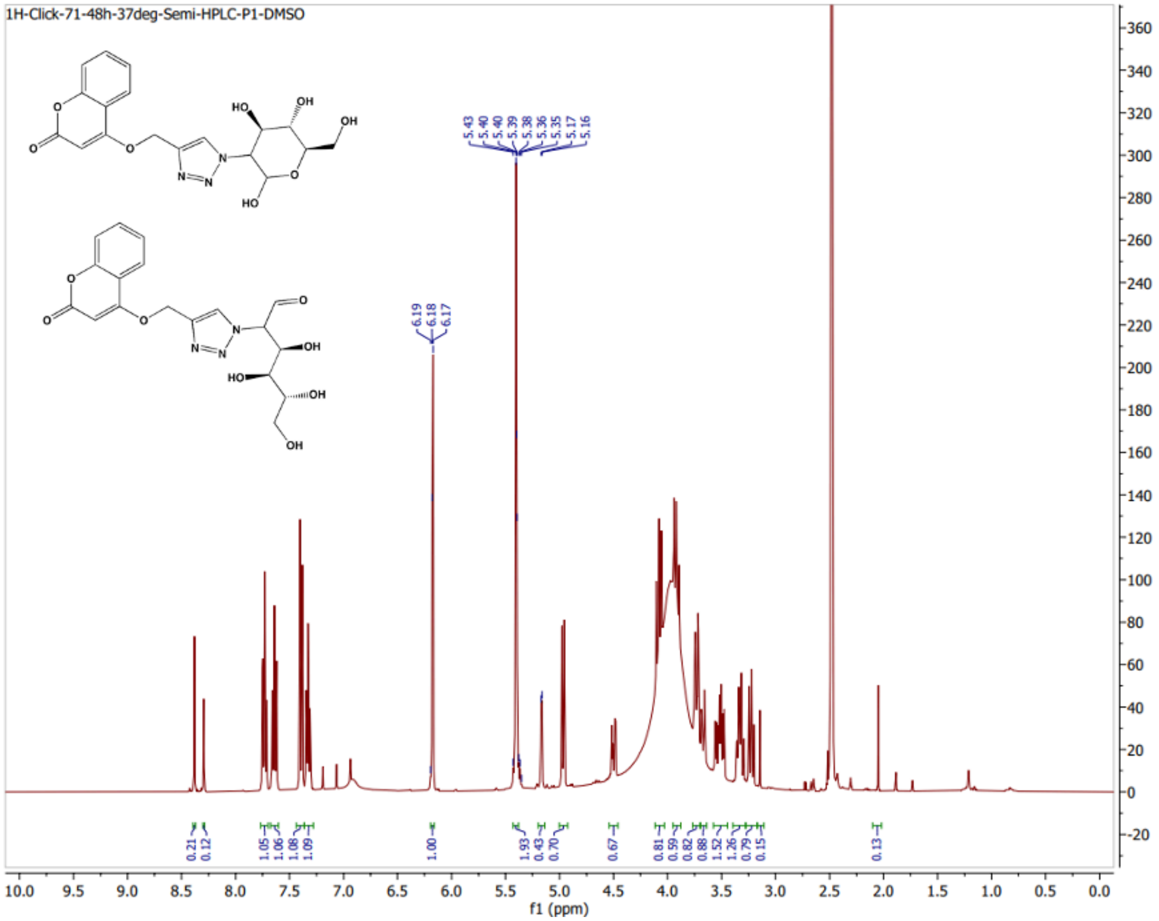

Method S- 4: Calculation of conversion to triazole 7.

The conversion to Triazole 7 is calculated by ^{1}H NMR spectrum of the crude mixture after removal of the solvents and dissolving the sample in d_6-DMSO. The integrated values for the newly formed triazole protons at 6.26 and 5.50 ppm were used to calculate the percentage yield with respect to the starting material in relation to the normalized integral of the remaining alkyne at 5.17 ppm (CH_2, normalized integral to 2.0) and the proton in the lactone ring of the alkyne at 6.05 ppm (CH, normalized integral to 1.0) were used (figure S-21) The normalized peaks were adjusted differently depending on whether they were compared to a single proton or two protons.

Figure S- 22: Determination of conversion to triazole 7 by ^{1}H-NMR.

A) integration of protons in crude reaction mixture; B) For percentage yield, the integrals of the triazole protons in positions 1 and 2 (product) was expressed as a percentage of the total integrals for the product and the starting material (normalized alkyne protons in positions a or b.

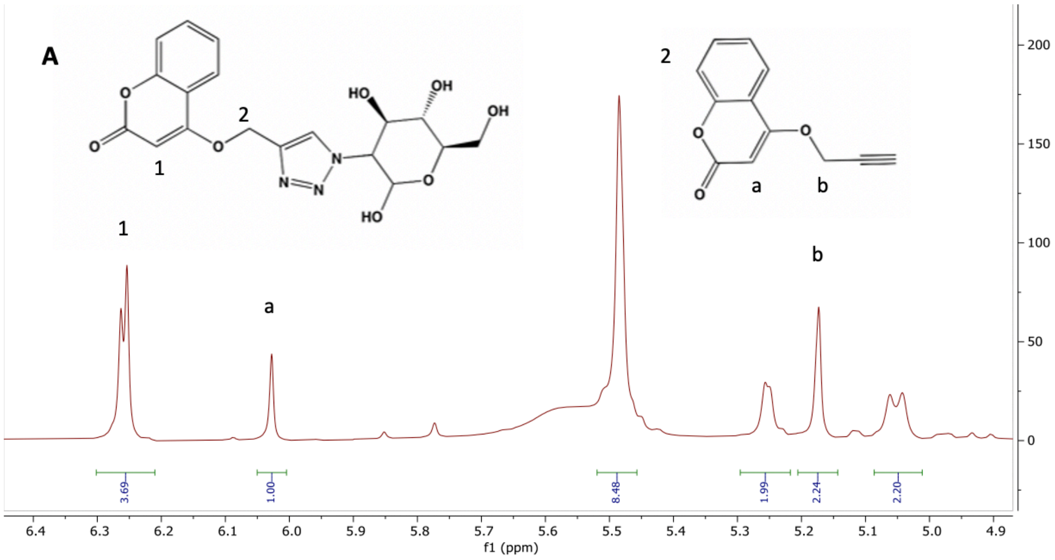

We integrated values of crude reaction mixture with remaining 4-(prop-2-yn-1-yloxy)-2H- chromen-2-one to calculate the conversion.

**6.22 ppm**	**6.00 ppm**	**5.45 ppm**	**5.14 ppm**
**tz-CH**	**CH-lactone**	**CH_2-lactone**	**CH_2-alkyne (b)**

**(1)**	**alkyne**<br><br>**(a)**	**tz**<br><br>**(2)**	
3.69	1.00	8.48	2.24
3.29	0.90	7.57	2.00

From the table, percentage yield 1 wrt position a = $\frac{3.69}{3.69+1}$*100 = 78.6 (when integral for proton at position a normalized to 1)

percentage yield 2 wrt position b = (8.48/10.72)*100 = 79.1 (when integral for proton at position a normalized to 1)

percentage yield 3 wrt position a = (3.29/4.19)*100 = 78.5 when integral for proton at position b normalized to 2)

percentage yield 4 wrt position b = (7.57/9.57)*100 = 79.1 when integral for proton at position b normalized to 2)

The average of those 4 values was reported as the percentage yield.

Figure S- 23: Monitoring of leaching of copper from resin using the absorbance of [Cu-(DDTC)$_2$]

A. Absorbance monitored for 1 μM to 50 μM [Cu-(DDTC)$_2$] solutions in DMF. B. The standard curve drawn has an R^2 of 0.96. C. On washing a previous version of the catalyst (not reported in this paper) bound to resin from the Cu(DDTC)2 calibration, it could be estimated how much nmoles of copper was removed in each DMF wash, after synthesis. After the 5th wash, no further change could be detected due to minimal formation of Cu(DDTC)$_2$. The catalyst was used only after 10-12 washes, and no Cu(DDTC) was found. For the new catalyst reported in this work, no formation of Cu(DDTC)$_2$ could be detected in the washes after the synthesis of the catalyst by monitoring the absorbance of Cu(DDTC)$_2$ at 475 nm.

A.

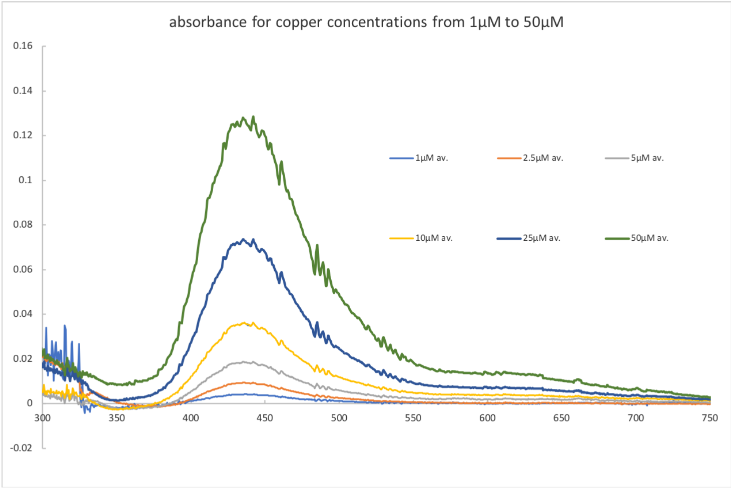

B.

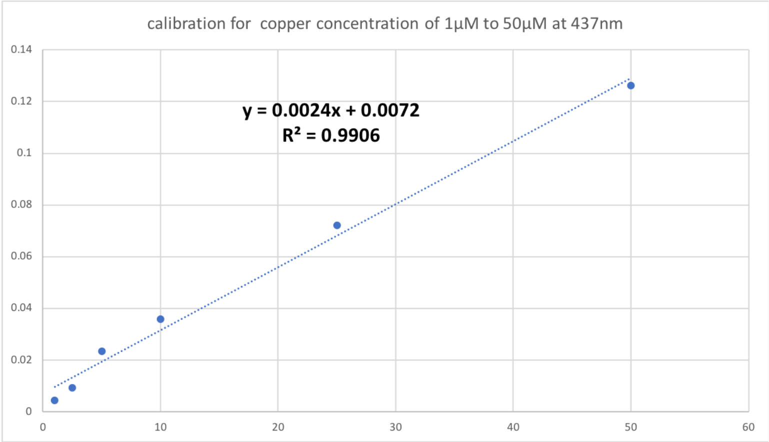

C.

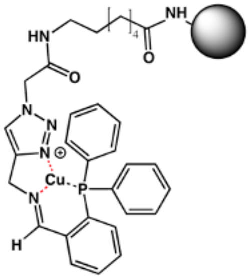

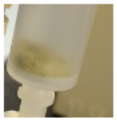

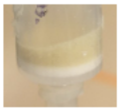

nmoles of copper

2000

300

45

12

0.5-3

List S- 2: Cartesian coordinates of the optimized structures A, B, P, LnCu, I1, I1’, I2, TS1, I3, TS2, I4

A

C -2.02252500 -0.00136400 -0.00036500
C -0.59297300 -0.00042200 0.00011500
C 0.11866800 1.21437700 0.00047400
C 0.11956900 -1.21475500 0.00041000
C 1.51166100 1.20996800 0.00000000
H -0.42892600 2.15128000 0.00114400
C 1.51261400 -1.20935800 0.00006700
H -0.42741100 -2.15203700 0.00071600
C 2.21213800 0.00055600 -0.00045400
H 2.05156300 2.15216700 -0.00015200
H 2.05327100 -2.15112800 0.00026600
H 3.29804200 0.00098600 -0.00106300
C -3.23341800 0.00066900 -0.00085100
H -4.30094500 0.00070300 0.00271500

B

N -2.23905900 -0.77503900 -0.11742800
N -2.36514000 0.39916100 -0.46865300
N -2.55724000 1.45166200 -0.87187100
C 0.17178900 -0.49943300 0.49791500
C 0.98418900 -1.25038400 -0.36187000
C 0.60474700 0.76471000 0.91568000
C 2.21148200 -0.74785300 -0.79244200
H 0.65066600 -2.23064600 -0.69249300
C 1.83348300 1.27019900 0.48509500
H -0.01874100 1.35165400 1.58529000
C 2.63811100 0.51481100 -0.36973400
H 2.83630300 -1.34019600 -1.45460200
H 2.16139400 2.25028900 0.81904100
H 3.59492200 0.90558000 -0.70366400
C -1.18085400 -1.01848300 0.91675100
H -1.49972000 -0.56656500 1.86275800
H -1.17243800 -2.10199700 1.04097000

P

C 1.33817700 -0.66038900 0.17938400
C 2.64235000 -0.00307200 0.04727700
C 2.91422500 1.20488600 0.71008600
C 3.64472600 -0.57686800 -0.75175500
C 4.15616400 1.82296300 0.57565500
H 2.15359700 1.66362700 1.33499800
C 4.88576700 0.04315200 -0.88376200
H 3.43843000 -1.50963100 -1.26539300
C 5.14781300 1.24543400 -0.22160000
H 4.34957200 2.75686500 1.09548300
H 5.65029100 -0.41360200 -1.50581600
H 6.11532100 1.72774900 -0.32531900
N -0.69396400 -1.24965100 0.65739900
N -0.17399300 -2.20945500 -0.13686100
N 1.05268900 -1.85821700 -0.42293300
C -2.95567900 -0.31200500 0.35952100
C -3.72212300 0.61042100 1.07625600
C -2.99376600 -0.29889500 -1.04080200
C -4.52667800 1.53383200 0.40311300
H -3.69025000 0.60740900 2.16262800
C -3.79159700 0.62506500 -1.71322700
H -2.39130900 -1.01050600 -1.59915000
C -4.56167200 1.54321800 -0.99176900
H -5.11900000 2.24676100 0.96918500
H -3.81479500 0.62931900 -2.79909000
H -5.18349800 2.26264200 -1.51650900
C 0.20612900 -0.26345100 0.87433000
C -2.09064500 -1.31974900 1.08534500
H -2.40471300 -2.34562000 0.87746400
H -2.12893300 -1.16270700 2.16588700
H -0.02697600 0.60170600 1.47408300

LnCu

C -1.15964100 0.50015000 2.92826400
H -1.50346000 1.47863900 2.56880000
H -1.13800700 0.55571600 4.03048200
N 0.16866600 0.27485300 2.36298000
Cu -0.49622200 1.72836300 -0.18886700
C 0.79305700 -0.96143000 2.82306000
H 1.01557900 -0.93667100 3.90458900
H 1.72595400 -1.12004200 2.27476800
C 1.06527800 1.42265300 2.55150100
H 1.62839000 1.36083000 3.49515100

H 0.46011800 2.33382400 2.59997300
C 2.00564400 1.53892300 1.39427300
C 3.36823200 1.40000700 1.27304700
H 4.14419200 1.18755900 1.99062600
C 4.88322400 1.47364600 -0.75663800
H 5.62929700 2.10259100 -0.26385900
H 4.72717100 1.85923800 -1.76488300
C 5.44887000 0.05913700 -0.85627200
C 5.25883700 -2.18313300 -0.15794600
H 4.53678200 -2.76246000 0.41329900
N 1.51940500 1.79283900 0.14207600
N 3.62000100 1.57756800 -0.05009700
N 2.49073200 1.81198800 -0.73570000
H 0.13487100 -1.80730000 2.62046700
H 6.24362600 -2.20887900 0.31279600
H 5.32848700 -2.55424300 -1.18196700
O 6.44681500 -0.17918900 -1.50120300
O 4.74951000 -0.83122700 -0.15302500
P -1.34742300 -0.32686100 -0.22473100
C -0.05696600 -1.60815000 -0.45106900
C -0.15546400 -2.89357500 0.09758200
C 1.05199800 -1.28548300 -1.24742300
C 0.84403400 -3.83857400 -0.14145600
H -1.00978500 -3.15880000 0.71110700
C 2.04342600 -2.23386600 -1.49398700
H 1.14270500 -0.29186100 -1.67387600
C 1.94326700 -3.51199600 -0.93826200
H 0.76029500 -4.83059300 0.29240600
H 2.89427600 -1.97156100 -2.11451500
H 2.71709700 -4.25015400 -1.12704000
C -2.43708400 -0.56728900 -1.68581200
C -3.36611800 0.44570600 -1.97397700
C -2.36945800 -1.69185900 -2.51965200
C -4.22296700 0.32828200 -3.06738000
H -3.41401100 1.32782900 -1.34059700
C -3.22233100 -1.80285000 -3.62012100
H -1.65453300 -2.48037000 -2.30965100
C -4.15069900 -0.79662000 -3.89411200
H -4.93988800 1.11616700 -3.27899200
H -3.16147400 -2.67733800 -4.26134000
H -4.81242000 -0.88531300 -4.75061000
C -2.37640000 -0.95001000 1.17452000
C -2.16739900 -0.55273500 2.51572700
C -3.38317300 -1.88648500 0.89009200
C -2.96178000 -1.12231700 3.51698000
C -4.17043200 -2.43690500 1.90095000

H -3.55442000 -2.19245000 -0.13579800
C -3.95711800 -2.05412500 3.22328500
H -2.79355500 -0.82435300 4.54812300
H -4.93951700 -3.16162600 1.65179200
H -4.55685700 -2.47730500 4.02341100
N -1.38903800 3.45856800 -0.32687800
C -1.97127900 4.45315200 -0.43309100
C -2.70560900 5.70099200 -0.56788000
H -3.27806000 5.89035800 0.34478900
H -2.00807900 6.52684200 -0.73388200
H -3.39363800 5.63324100 -1.41554300

I1

C -0.68337600 2.53956200 -2.32103000
H -1.09043400 1.69726400 -2.89517200
H -0.53662700 3.36803600 -3.03520300
N 0.58181600 2.09322400 -1.74059100
Cu -0.16422900 -0.60030400 -1.19752200
C 1.25075800 3.11527100 -0.93957600
H 1.56332600 3.98233100 -1.54771900
H 2.13627200 2.67964600 -0.46831200
C 1.48811900 1.53820200 -2.75353600
H 2.08965500 2.31530000 -3.25034900
H 0.88521400 1.05283700 -3.52832300
C 2.37952100 0.51566600 -2.12343600
C 3.74170300 0.42253400 -1.96343700
H 4.55609100 1.05585100 -2.27693600
C 5.15988900 -1.23502600 -0.67907800
H 5.96035200 -1.16742600 -1.41921000
H 5.00192200 -2.28875500 -0.44401500
C 5.61402800 -0.51333200 0.58827600
C 5.20353200 1.25324400 2.09100200
H 4.42686600 2.00358400 2.22013600
N 1.83110400 -0.56867200 -1.49634100
N 3.93061200 -0.71887800 -1.25037500
N 2.76587700 -1.31282100 -0.96031700
H 0.58458500 3.46315100 -0.14909800
H 6.17565400 1.71874100 1.91706800
H 5.25708300 0.59958800 2.96358200
O 6.61377300 -0.84823400 1.18537600
O 4.80871800 0.49410600 0.92636300
P -1.26318700 0.60699300 0.29100700
C -0.05696600 1.06290200 1.59733100

C -0.12132900 2.26193400 2.31926500
C 0.96681600 0.14554200 1.88093200
C 0.82567100 2.53849700 3.30715200
H -0.90401800 2.98282300 2.10948000
C 1.90328200 0.41751600 2.87683700
H 1.02948500 -0.78304500 1.32365700
C 1.83687500 1.61750100 3.58968200
H 0.77121600 3.47367700 3.85650300
H 2.68733900 -0.30315200 3.08767900
H 2.57086200 1.83412300 4.36020900
C -2.59915100 -0.26505700 1.19776400
C -3.68952700 -0.74134400 0.45256800
C -2.56083200 -0.49144500 2.57813900
C -4.73414400 -1.41333100 1.08243000
H -3.72598200 -0.57906000 -0.62139300
C -3.60341700 -1.17758200 3.20608000
H -1.72223800 -0.13172300 3.16439000
C -4.69237100 -1.63426100 2.46270500
H -5.57588200 -1.77154900 0.49710300
H -3.56423300 -1.34933800 4.27777300
H -5.50389900 -2.16319300 2.95364900
C -2.07484300 2.20034500 -0.16542900
C -1.70500200 2.97367000 -1.28988800
C -3.08454500 2.67746700 0.68779100
C -2.35125000 4.19756400 -1.50311000
C -3.72032000 3.89547000 0.45458100
H -3.37867600 2.08666700 1.54817900
C -3.35005700 4.66175000 -0.64854000
H -2.06195900 4.79609300 -2.36226900
H -4.49458900 4.23946700 1.13343500
H -3.83091700 5.61536900 -0.84420700
N -1.08059100 -1.86031500 -2.64752900
N -2.23333500 -1.55867700 -2.99920500
N -3.25119300 -1.17585100 -3.33572200
C -0.86891600 -3.51499700 -0.83737400
C 0.32508100 -3.40928400 -0.11072600
C -2.07477900 -3.71088000 -0.15304000
C 0.30931400 -3.48272600 1.28390500
H 1.26558700 -3.26245600 -0.63187300
C -2.08782700 -3.79226300 1.23839400
H -3.00605300 -3.78873400 -0.70743400
C -0.89825000 -3.66897200 1.95937900
H 1.23915000 -3.39503600 1.83828900
H -3.02846500 -3.92617600 1.76059400
H -0.91431900 -3.71756700 3.04393000
C -0.85579900 -3.31993600 -2.33238200

H -1.61552300 -3.91911400 -2.84011900
H 0.12232500 -3.55257300 -2.75612600

I1’

C 0.70174700 -0.83089000 2.81598600
H 1.14020300 -1.70363900 2.31519500
H 0.68817500 -1.06596200 3.89394500
N -0.64188400 -0.65498300 2.27447400
Cu 0.25643100 -1.55465400 -0.43199800
C -1.40716800 0.37304600 2.97496800
H -1.63888000 0.07513900 4.01233100
H -2.34315300 0.55742300 2.44071800
C -1.40025800 -1.91191600 2.20326400
H -1.98976400 -2.09507300 3.11365100
H -0.69432700 -2.74350500 2.10819400
C -2.29083100 -1.90877500 1.00184800
C -3.65477200 -1.96044000 0.83614700
H -4.47028300 -2.02414600 1.53803800
C -5.09392900 -1.86361000 -1.24839100
H -5.71436900 -2.71486800 -0.95491300
H -4.84910200 -1.97047500 -2.30568400
C -5.91697200 -0.59124300 -1.06264800
C -6.17958200 1.41522400 0.14684100
H -5.59630100 1.95964300 0.88645600
N -1.74601600 -1.81889600 -0.24766700
N -3.84520600 -1.89843100 -0.50772900
N -2.67973000 -1.80982000 -1.16431100
H -0.84154000 1.30463900 2.99769200
H -7.16460800 1.15444100 0.53887800
H -6.28832000 2.00085300 -0.76768100
O -6.92057200 -0.38719500 -1.71009100
O -5.42501000 0.21239800 -0.12015800
P 0.74027300 0.63483500 -0.13514400
C -0.74688300 1.69579500 -0.03331300
C -0.81414500 2.81404300 0.80754600
C -1.81660900 1.41852900 -0.89601700
C -1.94615600 3.63066500 0.80094100
H 0.01621000 3.04913700 1.46503300
C -2.94186700 2.24131800 -0.90837200
H -1.77018500 0.56459900 -1.56279400
C -3.01196900 3.34555800 -0.05521100
H -1.99141400 4.49190700 1.46080200
H -3.76074600 2.01824900 -1.58420500

H -3.88957800 3.98507400 -0.06338500
C 1.67172900 1.42214400 -1.51429300
C 2.73543600 0.71357700 -2.09268400
C 1.36099100 2.70095100 -2.00078300
C 3.48349500 1.27454600 -3.12636300
H 2.98451600 -0.27525000 -1.73311200
C 2.10656400 3.25796400 -3.04157500
H 0.54241600 3.26462700 -1.56765600
C 3.16981800 2.54901900 -3.60471100
H 4.30666200 0.71193900 -3.55689400
H 1.85584300 4.24840000 -3.41002500
H 3.74820600 2.98619700 -4.41324500
C 1.73867700 1.05096900 1.36112600
C 1.60773900 0.36565100 2.59434800
C 2.68079700 2.08610800 1.25634800
C 2.42066700 0.75303500 3.66552100
C 3.48895600 2.44918400 2.33315000
H 2.79159300 2.61441500 0.31689300
C 3.35932000 1.77770100 3.54595300
H 2.31615400 0.23170100 4.61280000
H 4.20994600 3.25275300 2.21916000
H 3.97742700 2.04853800 4.39641800
C 1.94655500 -2.85060400 -0.45360000
C 3.29222700 -2.34920000 -0.49037700
C 3.81372500 -1.59066300 0.57192100
C 4.07573800 -2.57420700 -1.63800300
C 5.08852700 -1.04166600 0.47436400
H 3.21335800 -1.41402100 1.45437000
C 5.35247900 -2.02349600 -1.72351400
H 3.66793600 -3.15615400 -2.45762600
C 5.85703100 -1.24957800 -0.67435500
H 5.47442800 -0.44071400 1.29154200
H 5.95001300 -2.19115500 -2.61400700
H 6.84651400 -0.81010700 -0.75243500
C 0.88633500 -3.48607000 -0.50844300
H 0.18242000 -4.29492100 -0.51044700

I2

C 1.55683100 -3.23156600 -0.93122900
H 1.90240300 -2.59629000 -1.75644600
H 1.75715700 -4.27420700 -1.23347100
N 0.13133100 -2.97124100 -0.76428600
Cu 0.51670000 0.07744300 -1.49586600

C -0.50765300 -3.88088600 0.18100900
H -0.52782700 -4.91879700 -0.19571500
H -1.53306800 -3.54966700 0.36700400
C -0.58460600 -2.95137600 -2.04481400
H -1.00616300 -3.93598400 -2.29673900
H 0.12571800 -2.70009100 -2.83888300
C -1.66580700 -1.91812400 -2.06095600
C -3.03244000 -2.00285800 -2.20526500
H -3.70275000 -2.84086700 -2.30482900
C -4.81232200 -0.18815900 -2.32405300
H -5.36186100 -0.79108100 -3.05247800
H -4.74219000 0.83122600 -2.69924100
C -5.59708500 -0.18210900 -1.01510600
C -6.15026200 -1.42689900 0.91131100
H -5.72954600 -2.28701500 1.42834400
N -1.35810600 -0.58909000 -1.97921700
N -3.46245000 -0.71442100 -2.20942000
N -2.44061200 0.14083600 -2.06961400
H 0.02905400 -3.86337800 1.13009900
H -7.18980500 -1.61046800 0.63200700
H -6.08913700 -0.53145500 1.53166100
O -6.38864300 0.69171000 -0.73249500
O -5.34969800 -1.26891700 -0.28256000
P 0.98233400 -0.43816300 0.66700300
C -0.45531400 -0.97811900 1.66454200
C -0.30759200 -1.81124100 2.78309800
C -1.72863000 -0.50558000 1.32596000
C -1.42044000 -2.18055900 3.53926000
H 0.67533500 -2.17750900 3.06034800
C -2.83786100 -0.87335600 2.08587400
H -1.85812500 0.16246800 0.48443700
C -2.69034800 -1.71591300 3.18865100
H -1.29417600 -2.83071900 4.40006900
H -3.81078800 -0.48303300 1.81904000
H -3.55808000 -2.00230800 3.77588000
C 1.57584900 1.03765000 1.59363300
C 2.49027900 1.88835800 0.95557500
C 1.12030100 1.37016100 2.87641500
C 2.93473800 3.05275500 1.57896500
H 2.85769600 1.64147100 -0.03251000
C 1.56484700 2.53714800 3.50145400
H 0.40825500 0.72886200 3.38282900
C 2.46706600 3.38368700 2.85373300
H 3.63836600 3.70055600 1.06478000
H 1.19986000 2.78684300 4.49358200
H 2.80413300 4.29476300 3.33916400

C 2.27089400 -1.71104900 1.03319700
C 2.38666400 -2.91397400 0.29598100
C 3.17069800 -1.47059700 2.08233200
C 3.38957400 -3.82449700 0.64577800
C 4.17282200 -2.38454800 2.40820600
H 3.09351000 -0.55216000 2.65189500
C 4.28374800 -3.56993600 1.68609900
H 3.47374000 -4.74894400 0.08117400
H 4.85584400 -2.16740600 3.22379800
H 5.05446700 -4.29510200 1.92897500
C 2.36883700 0.27500200 -2.73098100
C 3.64735200 0.48509900 -2.11380900
C 4.27863600 -0.54022300 -1.38834900
C 4.26241300 1.74862400 -2.20677700
C 5.48048000 -0.29306600 -0.73182800
H 3.81871900 -1.51625400 -1.32419700
C 5.46767400 1.98445400 -1.55028200
H 3.77986400 2.53743400 -2.77172000
C 6.07300100 0.97029900 -0.80237400
H 5.94454300 -1.08709100 -0.15576000
H 5.93063700 2.96396800 -1.61707800
H 7.00464300 1.16382500 -0.27977700
N -0.05714900 2.44859400 -1.39003200
N 0.88505700 3.22467000 -1.56899300
N 1.82865500 3.84541500 -1.73030500
C -2.06945600 2.87091400 0.00499800
C -3.43009400 2.54707300 0.05938800
C -1.35737600 3.03082400 1.19824100
C -4.07529700 2.39542100 1.28733200
H -3.98879100 2.41832200 -0.86198200
C -1.99933800 2.87202500 2.42655000
H -0.29775300 3.26231600 1.17079800
C -3.35864400 2.55624000 2.47517500
H -5.13012500 2.13846200 1.30852000
H -1.43166900 2.98703300 3.34443900
H -3.85405800 2.42545800 3.43272200
C 1.34397000 0.16233000 -3.40549100
C -1.40936900 3.07956300 -1.33724600
H -1.31499500 4.14715400 -1.56566000
H -2.00189500 2.60805300 -2.12141500
H 0.66876100 0.05457400 -4.23027100

TS1

C -1.18891000 3.26157800 -0.85322900
H -1.63439200 2.74629000 -1.71010800
H -1.26603200 4.34627900 -1.07019800
N 0.19602700 2.82205900 -0.76611600
Cu -0.42752000 -0.23164000 -1.50410700
C 0.97675100 3.60738700 0.18684200
H 1.10688800 4.64958400 -0.14728900
H 1.96579400 3.14867300 0.30169600
C 0.84434100 2.78276800 -2.07633500
H 1.30900300 3.74120700 -2.33577700
H 0.08754700 2.56494100 -2.83119500
C 1.85943600 1.68953800 -2.13104500
C 3.23255300 1.68237800 -2.19848800
H 3.96261000 2.48081000 -2.26380900
C 4.91542100 -0.21467100 -2.04714200
H 5.53796600 0.15510800 -2.86291000
H 4.80923700 -1.29408700 -2.11817000
C 5.53076600 0.13236900 -0.69255400
C 6.35141800 1.92628100 0.62627400
H 6.98119100 1.17164400 1.09926800
N 1.47251400 0.37831600 -2.03781800
N 3.58759300 0.37761300 -2.15765900
N 2.50637200 -0.41463200 -2.04858100
H 0.49459600 3.60480300 1.15914200
H 5.50689400 2.17974900 1.28438000
H 6.91681600 2.81793000 0.38243800
O 5.71129000 -0.68268400 0.19449600
O 5.82482700 1.43314300 -0.62934600
P -0.81238600 0.34796100 0.67923500
C 0.68907800 0.75358500 1.66614500
C 0.63029400 1.55414800 2.81182800
C 1.90481400 0.17048300 1.30174500
C 1.77202700 1.77338300 3.57484900
H -0.31543400 2.00848700 3.10505100
C 3.05800300 0.39571900 2.06218400
H 1.96103000 -0.48092100 0.43482700
C 2.99299700 1.19523800 3.20092400
H 1.71181500 2.39863400 4.46380100
H 3.99137200 -0.06383800 1.76167800
H 3.87698300 1.36427500 3.80428900
C -1.54729900 -1.06111100 1.60356700
C -2.50763800 -1.83986900 0.94663000
C -1.17522500 -1.40876600 2.90828900
C -3.09186200 -2.93886800 1.56908700
H -2.81095100 -1.58339700 -0.06217800
C -1.75682600 -2.51310800 3.54143600

H -0.42084800 -0.82343800 3.43215300
C -2.71185400 -3.28290600 2.87892700
H -3.83394500 -3.52712300 1.04167900
H -1.45108800 -2.77559800 4.55236400
H -3.15695100 -4.14703900 3.36974400
C -1.97998400 1.73781100 1.08590800
C -2.01469700 2.96548700 0.38433400
C -2.87195000 1.54866200 2.15681900
C -2.93456300 3.94923800 0.78472300
C -3.78675300 2.53681300 2.53552900
H -2.85899700 0.61593000 2.71220100
C -3.81064100 3.74235800 1.84398700
H -2.95352200 4.88315900 0.24035700
H -4.45241400 2.35802000 3.36807500
H -4.51191000 4.52667400 2.12116300
C -2.58189200 -0.51505100 -2.71496700
C -3.86403100 -0.34902300 -2.03547800
C -4.12164700 0.92305300 -1.47562600
C -4.80631300 -1.38377300 -1.82593000
C -5.23830200 1.14510200 -0.67576300
H -3.40933800 1.73060400 -1.65333500
C -5.93126600 -1.14508400 -1.04154700
H -4.69279400 -2.36179700 -2.28118100
C -6.13613200 0.09516200 -0.43848000
H -5.38839500 2.12319100 -0.22574800
H -6.64342100 -1.95558800 -0.88660500
H -7.00473900 0.25953300 0.20448300
N -0.19963900 -2.28607400 -1.77821200
N -1.31395700 -2.77448700 -2.19354200
N -2.37806500 -2.53179900 -2.62752300
C 1.56230200 -3.06562700 -0.19575300
C 2.92962500 -2.81983900 -0.09509400
C 0.78909200 -3.20378400 0.97066300
C 3.54066400 -2.72102700 1.15841500
H 3.52977700 -2.70867100 -0.99386800
C 1.39853200 -3.10069100 2.21533700
H -0.27101700 -3.37367700 0.89908800
C 2.77387300 -2.86281500 2.31738300
H 4.60444000 -2.50884100 1.22334800
H 0.80035000 -3.21474400 3.11806600
H 3.24548600 -2.78180300 3.30013900
C -1.54101400 -0.03169400 -3.21871600
C 0.93512600 -3.21781200 -1.55750800
H 0.57001900 -4.24579000 -1.70351600
H 1.67785600 -3.01337700 -2.33938600
H -0.90234500 0.12357400 -4.06763900

I3

C -1.68532100 -1.08341600 -2.40142100
H -2.21621700 -1.43702400 -1.50856100
H -2.05141400 -1.68322800 -3.24444700
N -0.26768000 -1.26865400 -2.17136000
Cu -0.43417700 -0.66696500 0.79106700
C 0.53123500 -1.04891500 -3.38235300
H 0.33360300 -1.80976400 -4.14823500
H 1.60115200 -1.07110700 -3.12622100
C 0.06361500 -2.57937300 -1.56843900
H 0.18918400 -3.37432500 -2.32056800
H -0.75438300 -2.87559000 -0.91648400
C 1.31358800 -2.40386000 -0.77373300
C 2.55912000 -2.99950300 -0.82487100
H 2.96694600 -3.77159100 -1.44366000
C 4.67242100 -2.53017100 0.49704600
H 4.89764000 -3.58097500 0.60003000
H 4.82751200 -2.02910500 1.45431300
C 5.64695100 -1.93899300 -0.53538900
C 5.89549200 -0.56845900 -2.43315900
H 5.22444000 -0.05272300 -3.10783900
N 1.36594200 -1.45599500 0.20280400
N 3.26909500 -2.35627100 0.14402000
N 2.54636500 -1.42991000 0.76636800
H 0.31118500 -0.07152000 -3.79347000
H 6.44637100 -1.35366200 -2.94730600
H 6.59374400 0.13878600 -1.96623800
O 6.83468200 -2.18376400 -0.49042300
O 5.05345800 -1.14835800 -1.41494400
P -0.32491200 1.29776600 -0.46051300
C 1.34476800 1.61321100 -1.07335900
C 1.60131300 1.97792000 -2.40189800
C 2.39006300 1.55082100 -0.13551200
C 2.91217300 2.24806600 -2.79989200
H 0.78992500 2.07207400 -3.12611500
C 3.69601900 1.81864000 -0.54502300
H 2.17359400 1.34641200 0.91899500
C 3.94875100 2.16744600 -1.88266100
H 3.11544400 2.55037500 -3.84064100
H 4.50647300 1.76317500 0.17629600
H 4.97049900 2.39818700 -2.19650300
C -0.71810500 2.76999000 0.56465600

C -2.02067200 2.97945000 1.04638500
C 0.27373600 3.71889300 0.89785400
C -2.32591900 4.04397400 1.86748500
H -2.82453500 2.32318700 0.73359300
C -0.03810600 4.80692900 1.71535600
H 1.28499600 3.60469900 0.54101500
C -1.32768100 4.96275700 2.22720200
H -3.33896000 4.17105300 2.23404300
H 0.74023000 5.52553700 1.95675700
H -1.56087200 5.80754400 2.86381600
C -1.49398300 1.45103100 -1.87997200
C -2.01974400 0.36766800 -2.63229500
C -1.84739000 2.76449600 -2.24522400
C -2.90925100 0.65036000 -3.67636200
C -2.71700300 3.02412000 -3.29438800
H -1.43160000 3.60628400 -1.69319500
C -3.27357200 1.96362200 -4.01049300
H -3.30581500 -0.16543900 -4.25920700
H -2.97628200 4.04822800 -3.54918700
H -3.96309100 2.13724500 -4.82971200
C -2.91237900 -1.77288700 0.97455100
C -4.31656900 -1.88644000 0.48035400
C -4.77132500 -0.95447300 -0.46618600
C -5.19770500 -2.86498000 0.95284900
C -6.08504600 -1.00131200 -0.93874100
H -4.08297700 -0.21838000 -0.86674500
C -6.50876300 -2.92097200 0.47453000
H -4.85075300 -3.59161300 1.69294400
C -6.95759500 -1.97465000 -0.47043400
H -6.41349500 -0.27224500 -1.67799200
H -7.19134000 -3.68511800 0.84154700
H -7.97152700 -2.02758600 -0.82805300
N -0.47516200 -1.99048100 2.14040200
N -1.34488800 -2.96256800 2.21394800
N -2.41881300 -3.00692700 1.49513900
C 0.91536500 -0.43068000 3.36809900
C 2.26139200 -0.06451600 3.51014600
C -0.06185400 0.58062600 3.46031900
C 2.63613900 1.26718700 3.68770700
H 3.02831800 -0.83573400 3.43967200
C 0.30375600 1.91101300 3.63418900
H -1.11761900 0.31090600 3.37968200
C 1.64799500 2.26877100 3.74235000
H 3.68221300 1.52638800 3.77130000
H -0.46700900 2.68094000 3.68124200
H 1.93372300 3.31028800 3.86371800

C -2.34037600 -0.53743400 0.90315900
C 0.54621500 -1.90028900 3.17261000
H 0.16986700 -2.34710000 4.10631500
H 1.44679800 -2.44869400 2.85898400
H -2.91076500 0.42531300 0.54630900

TS2

C -1.68532100 -1.07341600 -2.38142100
H -2.19621700 -1.41702400 -1.47856100
H -2.07141400 -1.68322800 -3.21444700
N -0.25768000 -1.27865400 -2.17136000
Cu -0.41417700 -0.66696500 0.75106700
C 0.53123500 -1.04891500 -3.38235300
H 0.32360300 -1.79976400 -4.15823500
H 1.60115200 -1.08110700 -3.13622100
C 0.07361500 -2.58937300 -1.58843900
H 0.18918400 -3.38432500 -2.34056800
H -0.74438300 -2.89559000 -0.92648400
C 1.32358800 -2.43386000 -0.79373300
C 2.57912000 -3.00950300 -0.84487100
H 2.99694600 -3.77159100 -1.47366000
C 4.68242100 -2.54017100 0.48704600
H 4.91764000 -3.59097500 0.60003000
H 4.82751200 -2.03910500 1.44431300
C 5.65695100 -1.93899300 -0.53538900
C 5.89549200 -0.56845900 -2.43315900
H 5.22444000 -0.05272300 -3.10783900
N 1.36594200 -1.49599500 0.20280400
N 3.27909500 -2.37627100 0.13402000
N 2.54636500 -1.45991000 0.76636800
H 0.31118500 -0.06152000 -3.78347000
H 6.44637100 -1.35366200 -2.94730600
H 6.59374400 0.13878600 -1.96623800
O 6.84468200 -2.18376400 -0.49042300
O 5.05345800 -1.14835800 -1.41494400
P -0.32491200 1.30776600 -0.46051300
C 1.34476800 1.63321100 -1.08335900
C 1.61131300 1.99792000 -2.41189800
C 2.39006300 1.56082100 -0.14551200
C 2.92217300 2.25806600 -2.80989200
H 0.79992500 2.09207400 -3.13611500
C 3.69601900 1.81864000 -0.54502300
H 2.16359400 1.34641200 0.90899500
C 3.95875100 2.16744600 -1.88266100
H 3.12544400 2.55037500 -3.85064100

H 4.50647300 1.75317500 0.17629600
H 4.98049900 2.38818700 -2.19650300
C -0.71810500 2.78999000 0.55465600
C -2.02067200 2.96945000 1.04638500
C 0.26373600 3.73889300 0.88785400
C -2.32591900 4.03397400 1.87748500
H -2.81453500 2.29318700 0.74359300
C -0.04810600 4.81692900 1.71535600
H 1.27499600 3.63469900 0.52101500
C -1.33768100 4.96275700 2.22720200
H -3.33896000 4.15105300 2.25404300
H 0.72023000 5.54553700 1.95675700
H -1.58087200 5.80754400 2.87381600
C -1.48398300 1.46103100 -1.88997200
C -2.00974400 0.37766800 -2.63229500
C -1.84739000 2.77449600 -2.25522400
C -2.90925100 0.65036000 -3.67636200
C -2.72700300 3.02412000 -3.30438800
H -1.43160000 3.61628400 -1.71319500
C -3.27357200 1.95362200 -4.01049300
H -3.30581500 -0.17543900 -4.24920700
H -2.98628200 4.04822800 -3.55918700
H -3.97309100 2.12724500 -4.82971200
C -2.92237900 -1.84288700 1.04455100
C -4.30656900 -1.88644000 0.51035400
C -4.74132500 -0.93447300 -0.42618600
C -5.19770500 -2.87498000 0.94284900
C -6.04504600 -0.97131200 -0.91874100
H -4.04297700 -0.17838000 -0.79674500
C -6.49876300 -2.91097200 0.45453000
H -4.86075300 -3.61161300 1.67294400
C -6.92759500 -1.95465000 -0.48043400
H -6.36349500 -0.23224500 -1.64799200
H -7.19134000 -3.67511800 0.80154700
H -7.94152700 -1.98758600 -0.85805300
N -0.68516200 -1.90048100 2.21040200
N -1.31488800 -3.02256800 2.12394800
N -2.39881300 -3.08692700 1.42513900
C 0.86536500 -0.39068000 3.35809900
C 2.22139200 -0.06451600 3.49014600
C -0.08185400 0.64062600 3.47031900
C 2.62613900 1.25718700 3.67770700
H 2.96831800 -0.85573400 3.40967200
C 0.31375600 1.96101300 3.64418900
H -1.14761900 0.40090600 3.41968200
C 1.66799500 2.27877100 3.74235000

H 3.68221300 1.48638800 3.76130000
H -0.43700900 2.75094000 3.70124200
H 1.98372300 3.31028800 3.86371800
C -2.26037600 -0.67743400 1.21315900
C 0.42621500 -1.83028900 3.18261000
H 0.08986700 -2.26710000 4.12631500
H 1.26679800 -2.41869400 2.79898400
H -2.70076500 0.30531300 1.29630900

I4

C -2.06533100 1.77142700 2.01470000
H -2.09164800 2.31170600 1.06409100
H -2.40667500 2.47926000 2.78733600
N -0.66613700 1.38631600 2.24636700
Cu -0.04426100 0.55574300 -0.11468200
C -0.47671900 0.61943100 3.48007000
H -0.72781500 1.21616200 4.37275200
H 0.56674000 0.30073700 3.54792800
C 0.20728600 2.57709900 2.24200200
H 0.39466900 2.94968700 3.25767500
H -0.30245400 3.37743500 1.69762400
C 1.50215800 2.30003200 1.54140700
C 2.81594800 2.61357100 1.80724800
H 3.29331800 3.10642700 2.63889600
C 4.94360800 2.03402300 0.53609100
H 5.44512500 2.77320100 1.16469600
H 5.17666500 2.24504800 -0.50727700
C 5.46443200 0.63981700 0.88175900
C 5.13106800 -1.27609100 2.21635400
H 4.47359300 -1.53979500 3.04244600
N 1.49293000 1.66058300 0.33294700
N 3.50871400 2.15086300 0.73104800
N 2.70494500 1.56364500 -0.15723500
H -1.10334700 -0.27229800 3.46692300
H 6.17141000 -1.23915000 2.54327500
H 5.02380400 -1.98842600 1.39625300
O 6.47535400 0.18215100 0.39501100
O 4.69611800 0.03264700 1.78786800
P -1.42630600 -1.06091600 0.33155500
C -0.59855400 -2.11283400 1.58632900
C -1.32742600 -2.93706900 2.45630400
C 0.80019100 -2.08726900 1.67531600
C -0.66440100 -3.72905900 3.39367600

H -2.41117800 -2.95489700 2.41026500
C 1.46128300 -2.88573100 2.60860600
H 1.37328500 -1.44561400 1.01479400
C 0.73065900 -3.70597800 3.47015800
H -1.23737400 -4.36200000 4.06450300
H 2.54486600 -2.86601600 2.66239100
H 1.24522100 -4.32236400 4.20115100
C -1.95854400 -2.22476600 -0.97375100
C -2.57451100 -1.69184200 -2.11649400
C -1.71045900 -3.60209000 -0.90162100
C -2.93699100 -2.52743500 -3.17083600
H -2.74694700 -0.62401100 -2.19643600
C -2.06682800 -4.43404900 -1.96529100
H -1.23892300 -4.02660300 -0.02245500
C -2.67928900 -3.89965900 -3.09992000
H -3.40814100 -2.10456300 -4.05301700
H -1.86503300 -5.49923400 -1.90374100
H -2.95292400 -4.54807700 -3.92689200
C -2.97994500 -0.53360500 1.16490100
C -3.08565900 0.64701300 1.94189300
C -4.07985000 -1.40371200 1.08054400
C -4.28872600 0.87686600 2.62403000
C -5.27078800 -1.14246300 1.75469000
H -4.00254000 -2.30051700 0.47562400
C -5.37207100 0.00382800 2.53881500
H -4.37856100 1.77441400 3.22903500
H -6.10375700 -1.83339200 1.66978800
H -6.28676500 0.22294800 3.08112800
C -0.86653500 2.01808800 -2.54296400
C -1.53505000 2.98033300 -1.66104400
C -0.80959400 3.99704600 -1.01798700
C -2.92011400 2.88882200 -1.44148700
C -1.45691200 4.90165500 -0.17655200
H 0.26007300 4.08432600 -1.18139600
C -3.56297800 3.79607800 -0.60141400
H -3.48114200 2.10166600 -1.93292100
C -2.83498700 4.80508800 0.03531000
H -0.88362400 5.68467800 0.31082300
H -4.63270500 3.70927100 -0.43673300
H -3.33616300 5.50884100 0.69258400
N 0.52121800 0.75426000 -3.63367600
N -0.72416300 0.40526300 -4.01492400
N -1.56266900 1.16970000 -3.36590800
C 2.16870800 -0.95157600 -2.97473200
C 3.46702400 -0.82894100 -2.47581400
C 1.31723400 -1.93424800 -2.44966400

C 3.92303000 -1.68621700 -1.47052000
H 4.12437900 -0.05926100 -2.87046400
C 1.77137200 -2.78914500 -1.44756400
H 0.30173600 -2.02781600 -2.82365500
C 3.07596200 -2.66819700 -0.95699100
H 4.93586600 -1.57926500 -1.09389600
H 1.10563400 -3.54577900 -1.04646300
H 3.42307800 -3.33802800 -0.17553900
C 0.48594300 1.74900600 -2.71656100
C 1.67844900 -0.02844100 -4.06991200
H 1.34110700 -0.58375200 -4.94801000
H 2.46298400 0.66502900 -4.38040900
H 1.38412000 2.16161000 -2.28810500
